# Nanosecond response perovskite quantum dot light-emitting diodes with ultra-high resolution for active display application

**DOI:** 10.1038/s41377-025-01959-y

**Published:** 2025-08-21

**Authors:** Qingkai Zhang, Kaiyu Yang, Chengyu Luo, Zhihan Lin, Weiguo Chen, Yongsheng Yu, Hailong Hu, Fushan Li

**Affiliations:** 1 College of Physics and Information Engineering, Fuzhou University, Fuzhou, China; 2 Fujian Science and Technology Innovation Laboratory for Optoelectronic Information of China, Fuzhou, China

**Keywords:** Quantum dots, Inorganic LEDs

## Abstract

Perovskite quantum dots light-emitting diodes (PeLEDs) have been developed for next-generation high resolution display applications. However, the hindered charge injection and massive charge trapping due to the insulating and defective surface of quantum dots (QDs) usually lead to a slow rise in electroluminescence (EL) response, which makes it challenging to realize ultra-high refresh rate displays with nanosecond response. Herein, an ionic liquid 1-Butyl-3-methylimidazolium Trifluoromethanesulfonate ([BMIM]OTF) was used to enhance the crystallinity and reduce the surface area ratio of QDs, which effectively decreases defect state and injection barrier at the interface. Therefore, the rise time of EL response with steady-state is successfully reduced by over 75%. We further reduce the capacitance effect by decreasing the light-emitting unit area. Thus, ultra-high resolution (9072 pixel per inch) PeLEDs with light-emitting pixel size of 1.3 μm were realized, achieving a brightness exceeding 170,000 cd/m^2^ and an external quantum efficiency up to 15.79%. Moreover, it achieves nanosecond ultrafast response time under steady-state, which is the fastest response time of PeLEDs reported so far. Our work represents the most advanced performance of ultra-high-resolution PeLEDs, and provides in-depth insights into the mechanism of improving their response speed, showing significant potential in high refresh rate active display application.

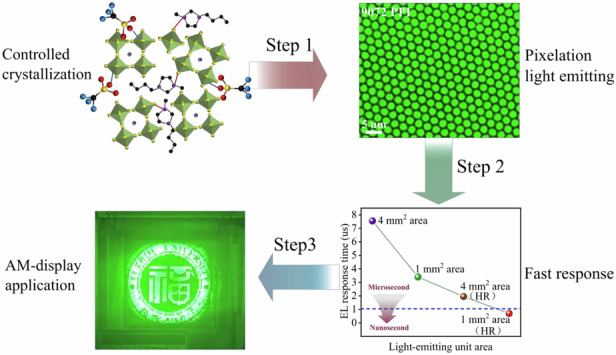

## Abstract (web summary)

Nanosecond-response Pe-QLEDs enabled by crystal-controlled and pixelated light emission of QDs

## Introduction

Lead halide perovskite quantum dots (QDs) have emerged as a promising material with a wide range of luminescent colors, narrow-band spectral purity, convenient synthesis methods, and excellent high-luminescence characteristics, showing tremendous potential for applications in high-performance optoelectronic devices, such as solar cells, detectors, lasers, especially in light-emitting diodes^1–5^. Currently, the external quantum efficiency (EQE) of red and green perovskite light-emitting diodes (PeLEDs) has been increased to over 20%^6–10^. Correspondingly, high-resolution PeLEDs for next-generation near-eye displays have also demonstrated obvious progresses^11,12^. For example, Liu et al. utilized carbene ligand-based photolithographic patterning to generate perovskite patterns with a resolution of 4000 pixel-per-inch (PPI)^13^. However, the efficiency of high-resolution light-emitting devices based on perovskite quantum dots is still insufficient for practical applications^14–19^. Moreover, none of these studies have addressed the response speed of the devices, which is crucial for future high refresh-rate display applications^20^. In addition, for more applications including visible light communication (VLC), the realization of high light fidelity requires devices with nanosecond response speeds^21^.

Generally, the response time is defined as the duration from the start of the pulse voltage to that of PeLEDs reaching 90% of the stable electroluminescence (EL) intensity^22,23^. The typical EL response time of PeLEDs shows two regions: an initial EL fast onset time upon application of the pulse voltage, followed by a slow rise before reaching the steady value^16,24–27^. According to previous reports, the slow rise process is related to defect density, as well as carrier injection and migration^2,27–30^. Although, nanosecond transient EL response has been reported in film based PeLEDs under high current density and short pulses^27,31^. However, the EL response with steady-state (under long pulse voltage) required for display is still above the microsecond level, including PeLEDs based on both film and quantum dots^31–35^. For example, Li et al. introduced a self-assembled monolayer [2-(9H-carbazol-9-yl) ethyl] phosphonic acid (2PACZ) to reduce the interface transport barrier, achieving 373 μs response time of film based PeLEDs^35^. Hou et al. used 3-(benzyldimethylammonio) propanesulfonate (3-BAS) to passivate quasi two-dimensional perovskite film defects, achieving microsecond response blue PeLEDs, and successfully applied in visible light communication systems^32^. Recently, Gao et al. demonstrated that the ion migration of the perovskite QDs layer would establish an internal electric field, which can affect the charge injection rate. Therefore, they fabricated a defect-free film with a discrete nanostructure and excellent crystallinity, successfully inhibiting ion migration and ultimately reducing the response time to tens of microseconds^36^.

In this work, we report the introduction of ionic liquid 1-Butyltrimethylimidazolium trifluoromethanesulfonic acid ([BMIM]OTF) to obtain perovskite quantum dots with enhanced crystallization and lower surface area ratio, which effectively reduce the defect states and increase the photoluminescence quantum yield (PLQY). Meanwhile, the introduction of [BMIM]OTF also promotes carrier injection at the interface, which contributed to a significant improvement in the response speed of the devices. As a result, the rise time of EL response with steady-state was significantly reduced by 75%, with the EQE of PeLEDs improved from 7.57% to 20.94%, and the T_50_ lifetime increased from 8.62 h to 131.87 h (converted to initial brightness L_0_ = 100 cd/m^2^). In addition, the ultra-high-resolution PeLEDs with pixel density of 9072 pixel-per-inch (PPI) and a light-emitting pixel size of 1.3 μm were fabricated, which successfully achieved ultra-fast response time of 700 ns under long pulse voltage, with a high brightness of 170,000 cd/m^2^, and a maximum EQE of 15.79%. In addition, we utilized QDs to construct and show a 64 × 64 pixel active-matrix display which exhibiting smooth image switching. Our work represents the most advanced performances of ultra-high-resolution PeLEDs, which is conducive to the development of fast-response high-resolution display technology.

## Results

**Fig. 1:**
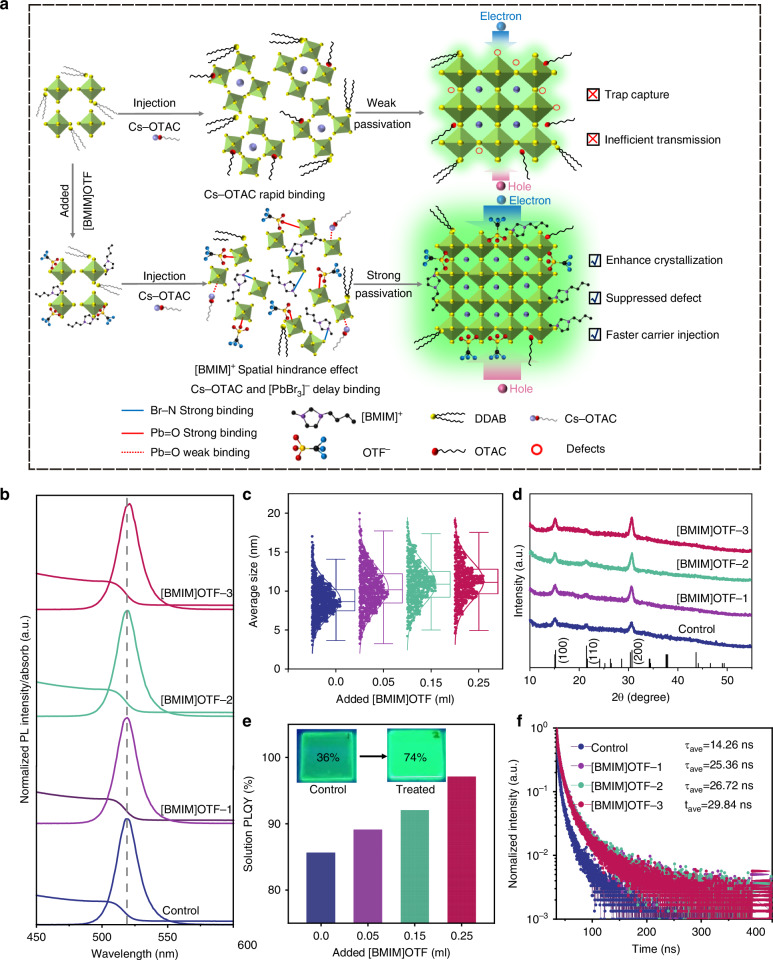
**The influence of [BMIM]OTF on QDs.** **a** The crystallization regulation process of QDs. **b** The PL and absorption graph. **c** The TEM size distribution of QDs. **d**, **e** XRD and PLQY graph. **f** TRPL spectra of QDs

The effect of [BMIM]OTF on the crystallization growth of QDs was first investigated. We adopted an in-situ crystallization strategy by adding [BMIM]OTF dissolved in chlorobenzene (CB) to the lead bromide precursor solution to control the nucleation process (Figs. 1a and S1). The addition amount of [BMIM]OTF was increased in sequence to obtain different types of QDs as Control, [BMIM]OTF-1, [BMIM]OTF-2 and [BMIM]OTF-3 (see Method for details). Since the positively charged N^+^ ions have a coordination effect with the Br^−^ ions, [BMIM]^+^ ions can combine with the [PbBr_3_]^−^ octahedron to form a complex^37–39^. Meanwhile, the imidazole ring of [BMIM]^+^ ions in the precursor solution exhibit steric hindrance effect^40^, which can delay the subsequent combination of Cs^+^ cations with [PbBr_3_]^−^ octahedrons, and slow down the nucleation process, thereby promoting the crystallinity and size growth of QDs^41–44^. According to previous reports, this result may be beneficial for carrier injection, because the low surface area ratio resulting from increased quantum dot size require less ligand passivation for QDs^45^. In addition, the close coordination of [BMIM]OTF with the surface of QDs can significantly suppresses surface defects and reduces surface charge trapping^46^. All of these results are expected to be conducive to the faster generation of excitons, thus quickly achieving the steady-state value of EL response^27^. Figure 1b shows the absorption and photoluminescence (PL) spectra of the as-synthesized QDs. The results show that the original emission peak of QDs is located at 517 nm, while with the increase of [BMIM]OTF, the PL peak red-shifts to 520 nm (Fig. S3a, b). In order to explore the reason of the red-shift, transmission electron microscopy (TEM) observation was performed and size distribution of the QDs was tested. As shown in Fig. S2a–d, all QDs showed excellent crystal morphology. The high-resolution TEM showed clear lattice fringes with a lattice spacing of 0.41 nm, corresponding to the (110) crystal plane of QDs^47^. The size distribution of QDs was careful statistically analyzed using nano-measurement software (Fig. 1c). The average grain size increased from 8.84 nm to 11.34 nm, confirming the effectiveness of [BMIM]OTF for enhancing the growth of particle size and the consequent red shift of the PL spectrum (Fig. S2e–h).

In order to further explore the origin of particle size growth, the crystallization of QDs was characterized by using X-ray diffraction (XRD) technique (Fig. 1d). All QDs exhibited the same monoclinic structure (PDF#18-0364), and the characteristic peaks were observed with no shift, indicating that the [BMIM]OTF did not enter the crystal lattice. In particular, the (200) crystal plane peak intensity was significantly enhanced, indicating that the [BMIM]OTF mainly interacted with the (200) crystal plane and enhanced the crystallinity of QDs along this crystal plane, resulting in obvious size growth^48^. Meanwhile, the PLQY of the QDs solution increased significantly from 85.6% to 97.1% (Fig. 1e), and the solid film deposited by [BMIM]OTF-3 treated also showed a higher PLQY value with a distinct bright green luminescence (Fig. S4c). Transient photoluminescence spectroscopy (TRPL) further illustrates the beneficial effect of [BMIM]OTF (Fig. 1f). After the addition of [BMIM]OTF, the exciton recombination lifetime (τ_*avg*_) increased from 14.26 ns to 29.84 ns. According to the three-exponential fitting calculation (Table S1), the increase of τ_*avg*_ indicates that the trap density is significantly reduced and the radiative recombination is therefore enhanced^49^. These results can be explained by the coordination of [BMIM]OTF which may suppresses the generation of surface defects during the crystallization process of QDs^39^.

**Fig. 2:**
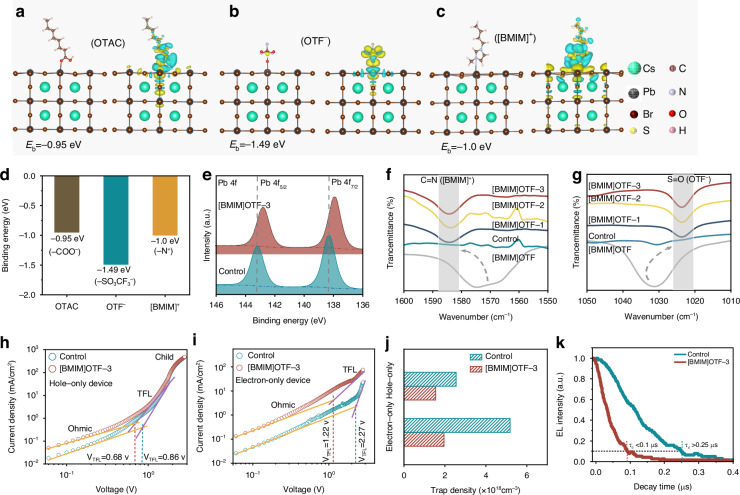
**Defect passivation of QDs by [BMIM]OTF.** **a**–**c** DFT calculation for binding energy and differential charge density image of QDs. **d** Comparison of binding energies between different ligands for QDs. **e** XPS spectra of Pb-4f. **f**, **g** FTIR spectrum of C=N and -SO_3_^−^ binding bond vibration. **h**, **i** Voltage–current density diagram of hole-only and electron-only devices. **j** Calculated trap density of control and [BMIM]OTF-3 devices. **k** TREL falling edge of devices

In order to further investigate the coordination effect of [BMIM]OTF, Density Functional Theory (DFT) calculation was carried out (see Supporting information for details). We compared the binding energies of [BMIM]OTF and the original ligand octanoic acid (OTAC) to QDs (Fig. 2d). The results show that the binding energy (E_b_) between the -COO^−^ of OTAC and the Pb^2+^ of QDs is −0.95 eV (Fig. 2a), which is much smaller than the E_b_ of −1.49 eV between OTF^−^ and Pb^2+^ (Fig. 2b), indicating that the passivation effect of OTF^−^ on the surface of QDs is stronger^50–52^. In addition, the binding energy of [BMIM]^+^ to Br^−^ on the QDs surface is −1.00 eV (Fig. 2c), indicating that the cations of [BMIM]OTF also have strong coordination to the surface of QDs. The distribution of charge density difference between the QDs surface and the three functional groups was further calculated to study the charge transfer between QDs and OTAC along with [BMIM]OTF ligands (Figs. 2a–c and S5a–d). The electron depletion around Pb^2+^ ions (blue cloud) and the electron accumulation around O atoms from carboxyl group of OTAC or sulfonic acid group of OTF^−^ (yellow cloud) indicate the interaction between Pb^2+^ and OTAC or OTF^−^^52^. In comparison, [BMIM]^+^ and Br^−^ have more electron clouds distributed around the entire molecule, indicating that the conductivity of the ionic liquid [BMIM]OTF mainly depends on the cation [BMIM]^+^ with an imidazole ring. The highly conductive [BMIM]^+^ cation may provide an effective charge transfer path, thereby improving the carrier transport and injection of QDs layer^46^.

To further confirm the coordination of [BMIM]OTF on the QD surface, the binding energy of QDs were studied by X-ray photoelectron spectroscopy (XPS). In the Pb-4f spectrum, the binding energy of Pb-4f for the control quantum dots is at 138.29 eV and 143.16 eV. While after adding [BMIM]OTF, the Pb-4f peak shifts to 137.86 eV and 142.74 eV (Fig. 2e). The decrease in the binding energy of Pb-4f indicates a reduction in the cationic charge around the Pb^2+^ ion, which can be attributed to the strong coordination between the lone electron pair of the S = O bond in [BMIM]OTF and the Pb^2+^ cation, resulting in a reduction in the surface dangling bonds on the Pb^2+^ cation and an effective passivation of the Pb^2+^ cation^39^. A similar trend was also observed in the Cs-3d spectrum (Fig. S6a). In addition, shift was also found in the Br-3d spectrum (Fig. S6b), which can be attributed to the binding of [BMIM]^+^ and Br^−^^47^. The coordination of [BMIM]OTF and QDs were further verified by analyzing Fourier transform infrared spectroscopy (FTIR). Two peaks were observed at 1584 cm^−^^1^ and 1023 cm^−^^1^, corresponding to the stretching vibrations of C = N (from [BMIM]^+^ cation) and S = O (from OTF^−^ anion), respectively (Fig. 2f, g)^53,54^, consistent with DFT calculations and XPS results. In addition, the vibration at 1716 cm^−^^1^ is attributed to the stretching vibration of the C = O bond (Fig. S7b), indicating that all QDs contain the original OTAC ligands. Similarly, the original Didodecyldimethylammonium bromide (DDAB) ligand provides C-N stretching vibrations near 966 cm^−^^1^ and 915 cm^−^^1^ (Fig. S7c)^53^.

In order to investigate the effect of [BMIM]OTF on the carrier transport and defects suppression of QDs, hole-only and electron-only devices were prepared, and analyzed based on space charge limited current (SCLC) model^55,56^. The hole-only device has a structure of indium-tin-oxide (ITO)/ poly(3,4-ethylenedioxythiophene) poly(styrenesulfonate)(PEDOT:PSS)/poly(3,4-ethylenedioxythiophene)poly(styrenesulfonate)‌(PTAA)/QDs/MoO_3_/Al, while the structure of electron-only device is ITO/ 1,3,5-Tris(1-phenyl-1H-benzimidazol-2-yl) benzene (TPBi)/QDs/TPBi/LiF/Al (Fig. S8a, b). As shown in Fig. 2h, i, the current of both hole-only and electron-only devices has increased, proving that the treatment of [BMIM] OTF improves the carrier transport capability of QDs layer. In addition, the trap-filled limit voltage decreased from 0.86 V to 0.68 V for the hole-only device, and from 2.27 V to 1.12 V for the electron-only device. The results show that after the addition of [BMIM]OTF, the trap density of the hole-only device decreased from 1.96×10^18 ^cm^−3^ to 1.55×10^18 ^cm^−3^, and the trap density of the electron-only device decreased from 5.18×10^18 ^cm^−3^ to 2.55×10^18^ cm^−3^ (Figs. 2j and S8c, d). The decrease in the film trap density can effectively reduce the charge trapping of QD layer, which is beneficial to faster carrier injection of QDs. The result was further verified by the decay rate of the falling edge of time-resolved electroluminescence (TREL), in which the decrease in the TREL of the [BMIM]OTF-3 device was significantly faster than that of the control device based on pristine QDs (Fig. 2k). Because the decay of the falling edge was caused by the recombination of the carriers trapped by the defects, the faster rate of decline indicated that the [BMIM]OTF device had lower defect state density, which is consistent with the previous results^27^. All the above results demonstrate that [BMIM]OTF has a strong binding ability to QDs, which can effectively suppress their surface defects and reduce charge trapping.

**Fig. 3:**
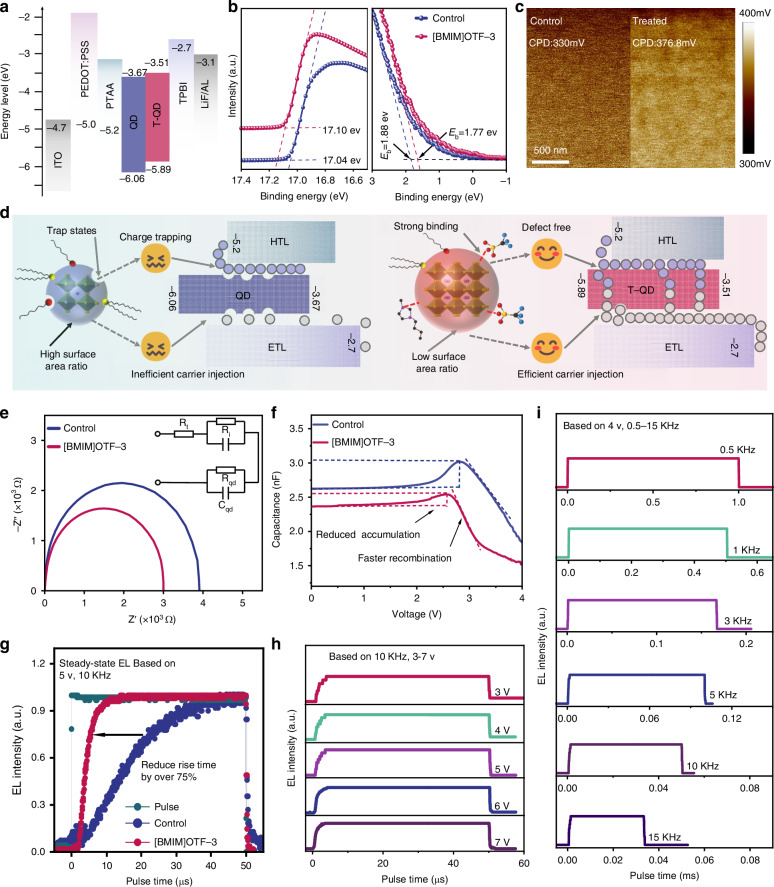
**The dynamics of carrier transport and injection in devices.** **a** The diagram of energy level structure. **b** UPS of QDs films. **c** KPFM images of Control and [BMIM]OTF-3 QDs film. **d** Schematic diagram of charge transfer and injection between QDs and the transport layers. **e** The impedance spectrum of the devices. **f** The capacitance-voltage curve of the devices. **g** TREL spectra of control and [BMIM]OTF devices. **h**–**i** TREL spectra with different voltages and frequency

The effect of [BMIM]OTF on charge injection was further investigated to study its influence on EL response of PeLEDs. From the voltage-current curve of single-carrier devices, we can see that the transport of both holes and electrons has been improved, especially the electrons (Fig. 2i). This result facilitates the rapid injection of mass electrons and their recombination with holes, thereby enabling the EL intensity to quickly reach its maximum value. To explain the obvious improvement of the electron transport, ultraviolet photoelectron spectroscopy (UPS) and Kelvin Probe Force Microscopy (KPFM) observations were carried out. The work functions of pristine and [BMIM]OTF-3 QDs films are estimated to be −4.18 eV and −4.12 eV respectively (Figs. 3b and S9a, b). The reduction of the work function after [BMIM]OTF addition favors the electron transport of QDs film, which is consistent with previous reports^54^. Meanwhile, we further calculated the bandgap of QDs based on the absorption curve (Fig. S9c), the valence band of the QDs film is increased from −6.06 eV to −5.89 eV, and the corresponding conduction band is increased from −3.67 eV to −3.51 eV (Figs. 3a and S9b). The overall upward shift of the energy level of the QDs film matched better with the energy levels of the transport layers, thus reducing the injection barrier and charge accumulation at the interface, which is conducive to the faster injection of both holes and electrons^29^. The KPFM observation further confirmed that the improvement in electron transport resulted from the reduction of the work function of QDs film. After adding [BMIM]OTF, the surface potential of the [BMIM]OTF-3 QDs film increased significantly from 330 mV to 376.8 mV (Fig. 3c). As the surface potential becomes higher, the corresponding work function decreases, which is consistent with the UPS results.

In order to further verify the improvement of charge injection capability and the reduction of charge trapping after [BMIM]OTF treated, we conducted electrochemical impedance tests of the control and [BMIM] OTF-3 devices at 1 V voltage, across the frequency range of 20 Hz–10 KHz, and using the PeLEDs equivalent-circuit model as shown in Fig. 3e. In this model, the QDs layer is represented as a parallel circuit comprising a capacitor C_*qd*_ caused by charge trapping and resistor R_*qd*_. Furthermore, due to the charge injection barrier and accumulation, the interface of QDs layer is represented as a parallel circuit of barrier capacitance C_i_ and resistance R_i_. The Nyquist plot analysis shows that the addition of [BMIM]OTF reduces both the real and imaginary parts of the impedance of PeLEDs. The decrease of the impedance can be explained by the [BMIM]OTF which can passivate defects and regulate energy level of QDs layer, thus reduces the capacitance (caused by charge trapping and accumulation) and resistance of the device. To further verify the reduction of charge trapping and interface injection barrier of QDs layer, the capacitor-voltage characteristic (C-V) of device was tested. As shown in Fig. 3f, the addition of [BMIM]OTF effectively reduces the capacitance of PeLEDs near the turn-on voltage. Because the increase of capacitance is mainly caused by charge trapping of QDs layer and charge accumulation at the interface, this results further proof that [BMIM]OTF can passivate defects and regulate energy levels of QDs layer, thus reducing capacitance caused by charge trapping and energy barrier. In addition, the more significant falling of C-V curve in the recombination region also indicates an increased injection of unbalanced electrons^57^. Based on the above analysis, we established hole and electron transport models of PeLEDs, as shown in Fig. 3d. The original QDs layer will capture holes and electrons due to its abundant interface defects. In addition, at the beginning, very few electrons can recombine with holes as the result of unbalanced electrons injection. After passivation with [BMIM]OTF, defects are effective reduced and the trapping of charges at the interface is suppressed. Meanwhile, due to the improvement of energy levels and transport capability of QDs layer, which cause more electrons and holes to quickly meet, the luminescence intensity (EL intensity) can rapidly reach its maximum steady value under stable voltage, thus improving the EL response speed of the devices.

To further characterize the response speed of the device, we tested the EL response with steady-state (TREL based on low voltage and low-frequency pulse) of the control and [BMIM]OTF modified devices at the same voltage (5 V) and frequency (10 KHz). As shown in Fig. 3g, the rise time of steady-state EL response for the [BMIM]OTF-3 device was significantly decreased from 32.3 μs of the control device to 7.55 μs, with more than 75% reduction (Figs. 3g and S10a). Therefore, we have successfully demonstrated the great potential of introducing [BMIM]OTF in improving the EL response speed of PeLEDs. In addition, we try to further reduce the influence of time constant (τ = RC, where τ is the time constant, and R and C are the equivalent resistance and capacitance of the device, respectively) on the response speed of PeLEDs. According to the formula ${\rm{C}}=\frac{{\rm{\varepsilon }}{\rm{S}}}{4{\rm{\pi }}{\rm{kd}}}$ (Assuming it is a simple parallel plate capacitor, where ${\rm{\varepsilon }}$ is dielectric constant, k is electrostatic force constant, S and d represent the area and thickness of the capacitance, respectively), we can reduce the equivalent capacitance of the device by reducing its area^58^. Therefore, we further decreased the light-emitting area of the device from 4 mm^2^ to 1 mm^2^ (Fig. S10b, d). As expected, the response time of the device is further decreased to 3.4 μs (Fig. S10c). In addition, Fig. S10e shows that the front and rear pulse pulses have no significant effect on the response time. We also investigated the response of [BMIM]OTF devices with 1 mm^2^ light-emitting area at different voltages (based on 10 KHz, 3 v to 7 v). The response time is reduced from 3.75 μs to 2.25 μs (Fig.3h), in which the onset time and rise time of EL intensity were both slightly shortened (Fig. S10f). Therefore, the decrease in response time can be attributed to the higher voltage, which makes it easier to overcome the interface barrier and accelerates trap filling of the QD layer. As shown in Fig. 3i, we also tested the EL response time with different frequencies (based on 4 v, 0.5 KHz to 15 KHz). The response time of all frequencies maintain at 3.4 μs, indicating that different long pulse frequencies have no obvious effect on the device steady-state EL response under low voltage (Fig. S10g).

**Fig. 4:**
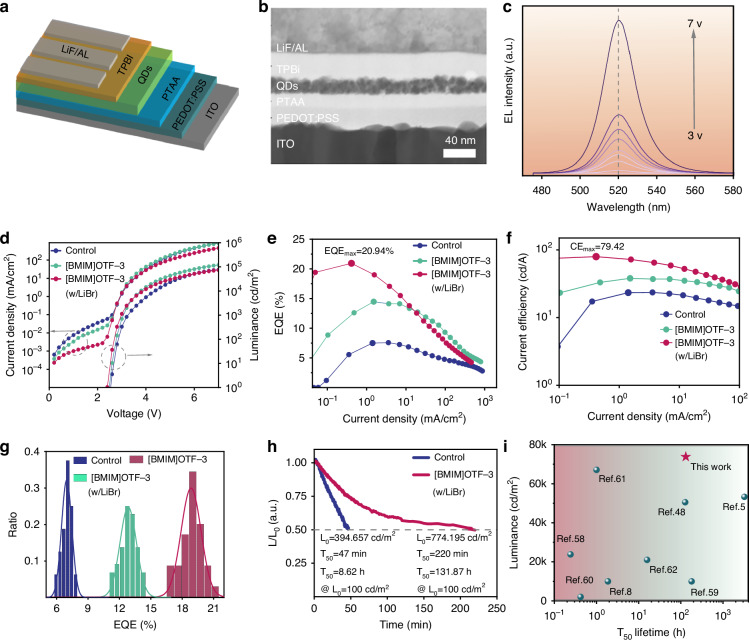
**Performance of PeLEDs devices.** **a** Diagram of the device structure. **b** SEM image of device cross-section. **c** Stable EL spectra at different voltages. **d**–**h** J-V-L characteristics, EQE, Current efficiency, EQE distribution, and lifetime of the devices. **i** Lifetime and brightness comparison scatter plot of green perovskite quantum dots light-emitting diodes

**Fig. 5:**
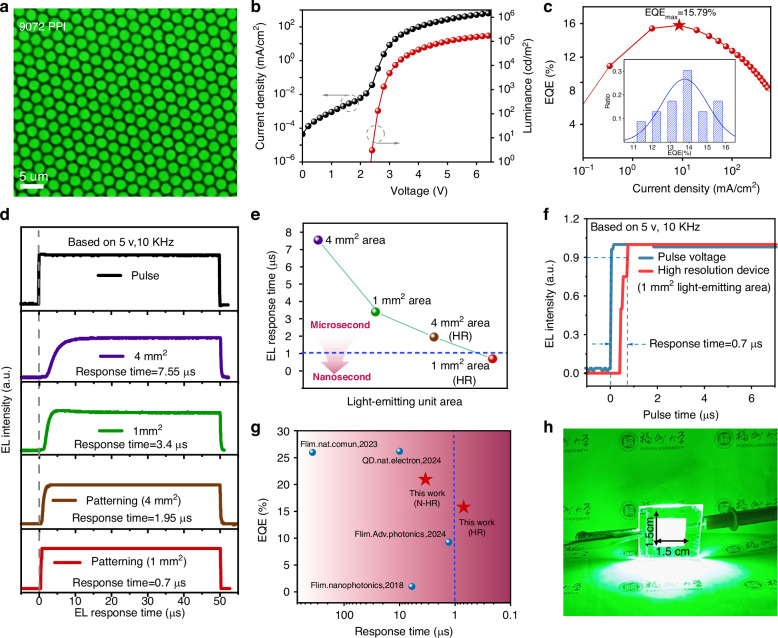
**The application in high-resolution PeLEDs.** **a** Photoluminescence image of high-resolution QDs film. **b** J-V-L and **c** EQE of high-resolution devices, inset shows the distribution of EQE. **d** TREL plots of devices with different light-emitting unit areas. **e** Diagram of the relationship between response time and light-emitting unit area. **f** TREL plot of high-resolution device with a light-emitting area of 1 mm^2^. **g** Comparison of the reported PeLEDs on the basis of steady-state EL response time and EQE. **h** Photograph of large area device (2.25 cm^2^)

The influence of [BMIM]OTF on the device performance was then systematically investigated. The device structure of PeLEDs is ITO/PEDOT: PSS/PTAA/PQDs/TPBi/LiF/Al, as shown in Fig. 4a. The cross-sectional image of the device shows smooth and clear interfaces between each layer, which is conducive to the carrier injection to achieve efficient PeLEDs (Fig. 4b). Figure 4c shows that the [BMIM]OTF-3 device has a stable electroluminescent (EL) emission at 520 nm with narrow half peak width, which is suitable for green display (Fig. S11a, b). As shown in Fig. S11c, d, the efficiency increases as the addition amount of [BMIM]OTF and reaches the peak value in the device based on [BMIM]OTF-3. However, when an excess concentration of [BMIM]OTF is added, the current density of the device increases significantly, accompanied by a significant decrease in the device efficiency. This result can be attributed to the poor morphology of QDs film with excessive addition of [BMIM]OTF, resulting in large leakage current, which can be confirmed by the atomic force microscopy (AFM) image of the QDs films (Fig. S12e). As shown in Fig. 4d, e, with the optimal addition amount of [BMIM]OTF, the EQE of the devices increased significantly from 7.56% to 14.47%, with more than 91% enhancement, indicating that the addition of [BMIM]OTF has a very positive impact on device performance. In order to further improve the device efficiency, we also added an ultra-thin lithium bromide layer with thickness of 2 nm at the interface between hole transport layer (PTAA) and QDs layer^59^, the TEM image of the device structure is shown in Fig. S13a. As shown in Fig. 4d, the introduction of lithium bromide further significantly increased the device efficiency to 20.94% (Fig. 4e). It is worth noting that the peak current efficiency of the device is also significantly increased by 2.73 times, from 23.55 cd/A to 79.42 cd/A (Fig. 4f). Meanwhile, we also investigated the effect of lithium bromide introduction on response speed. As shown in Fig. S13b, c, with the introduction of lithium bromide, the response time was shortened from 7.55 μs to 4.9 μs. This may be attributed to the additional defect passivation by lithium bromide, which further reduces the charge trapping at the interface between PTAA and QDs layer. Figure 4g shows the EQE distribution of the devices, and the statistical analysis of 18 devices confirmed their good reproducibility. The T_50_ lifetimes of the devices were further tested at a same current density of 3 mA/cm^2^, as shown in Fig. 4h. Setting the acceleration factor *n* = 1.75, the T_50_ lifetime of the devices can be calculated to be 8.62 h and 131.87 h (converted to the same initial brightness of L_0_ = 100 cd/m^2^), respectively (Fig. S13d). Therefore, the operation stability of the device is significantly improved by 14 times, which can be attributed to the strong interaction between [BMIM]OTF and QDs. As shown in Fig. 5i, compared with previous green PeLEDs, our device exhibits the highest brightness and excellent lifetime, which can well meet the display requirements^5,8,50,60–64^. In addition, in order to verify the universal applicability of [BMIM]OTF for the improvement of device performance, we also prepared a pure blue PeLEDs (Fig. S14a, b). The corresponding brightness and efficiency of the device are also significantly improved by 78% and 42%, respectively (Fig. S15c, d).

Inspired by the effect of reducing device area on improving response speed, we further investigated the ultra-high resolution PeLEDs with microscale pixel area, which were prepared by using nanoimprinting technology^65^. The high-resolution device structure is shown in Fig. S15a, with an ultra-high resolution of 9072 PPI, the leakage current between pixels is effectively blocked by honeycomb shaped polymethyl methacrylate (PMMA) (Fig. S15d). The two-dimensional structure of PDMS stamp and sacrificial layer HPC is shown in Fig. S15b, c. In addition, we determined the depth of PMMA through AFM measurement (Fig. S15e), and a depth close to 100 nm effectively separated the quantum dot pixels (Fig. S15f–g), enable in a neat pixel arrangement in the luminescent layer (Figs. 5a and S15i). The resulted high-resolution PeLEDs with the addition of [BMIM]OTF-3 exhibited a high brightness of 171547.13 cd/m^2^, a peak EQE of 15.79% (Figs. 5b, c and S16f), a response speed of 1.95 us (Fig. S16h), and based on an initial brightness of 100 cd/m^2^, the T_50_ lifetime exceeds 74.4 h (Fig. S16g). In addition, in order to demonstrate the luminescence uniformity of display pixels, we conducted a statistical analysis on the electroluminescence. The electroluminescence photos, pixel segmentation maps, luminance statistical histograms, and luminance spatial heatmaps are shown in Fig. S16a–d, and the results indicate that the pixels have excellent luminance uniformity of 86.13% (Fig. S16e). As shown in Fig. 5d, we further explored the response time of the devices with different light-emitting areas, including prototype devices with light-emitting areas of 4 mm^2^ and 1 mm^2^, and high-resolution (9072 PPI) devices with light-emitting areas of 4 mm^2^ and 1 mm^2^. As detail shown in Fig. S17c, the response time are 7.55 μs, 3.4 μs, 1.95 μs and 0.7 μs, respectively (based on 5 V, 10 KHz). It can be seen that as the area of the light-emitting decreases, the response speed of the device increases accordingly (Fig. 5e). This is because the size of the light-emitting area can affect the equivalent capacitance of the device, which further reduces the charge accumulation at the interface as discussed previously, and ultimately realizes device response with nanosecond level. Further, we using “Zview” software fitted the impedance spectrum of a 1 mm^2^ light-emitting area device to further demonstrate that the capacitance effect is suppressed by reducing the light-emitting area of QDs. Compared to the prototype device, the high-resolution patterned device exhibits a significant decrease in both resistance and capacitance (Fig. S17a, b). The EQE and resolution of our devices, especially the response time of 700 ns (Fig. 5f), represent the best performance of high-resolution PeLEDs reported so far (Fig. 5g, Tab. S2, S3, Fig. S16i). In addition, we also demonstrated that [BMIM]OTF modified QDs ink has high reliability in the preparation of large-area (2.25 cm^2^) devices (Fig. 5h) and wearable flexible devices (Fig. S17d–f).

**Fig. 6:**
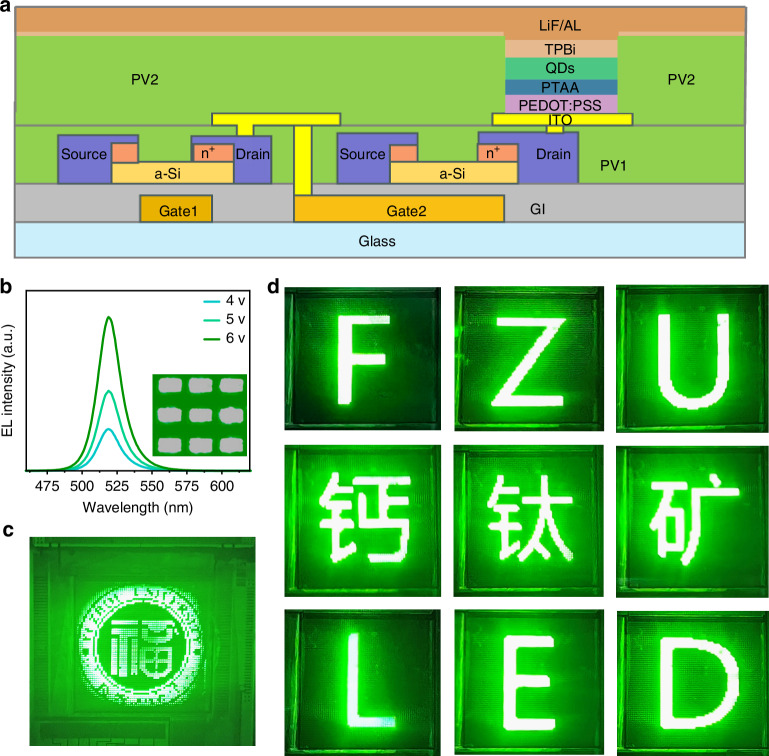
**The application in AM-PeLEDs.** **a** AM-PeLEDs display structure diagram. **b** PL spectroscopy and microscopic photos of AM-PeLEDs. **c** The LOGO of Fuzhou University. **d** Multiple images displayed, including letters “FZU” and Chinese names for perovskite, as well as letters “LED”

Finally, as our PeLEDs exhibit ultra-fast response capabilities, they are highly suitable for high-refresh-rate active-matrix displays of perovskite LED. To further verify the display performance, we fabricated and showed an active-matrix PeLEDs (AM-PeLEDs) on an independently drivable TFT substrate, with active dimensions of 17.28 × 17.28 mm^2^ and a 64 × 64 pixel-resolution, emitting bright green light (Fig. 6a, b). Based on the AM-PeLEDs, we demonstrated various images, including the LOGO and initials “FZU” of Fuzhou University, the Chinese name of perovskite, the letter “LED” and the cartoon character of Xiao Zhi (Figs. 6c, d, and S18a–i). More importantly, it displayed smooth dynamic image switching (Videos 1–3), highlighting potential of our fast response PeLEDs in the future display application.

## Discussion

In conclusion, we have provided a promising strategy for improving the EL response of devices by adding ionic liquid [BMIM]OTF and reducing light-emitting unit area for QDs, with ultra-high resolution PeLEDs successfully achieved nanosecond response time. The results indicate that [BMIM]OTF can significantly suppress charge trapping and accumulation by reducing defect and interface injection barrier of QDs layer. As a result, the rise time of the EL response of PeLEDs is shortened by over 75%, with a T_50_ lifetime of 131.87 h and an external quantum efficiency of 20.94%. In addition, the high-resolution device with a pixel density of 9072 PPI exhibits a high brightness of 171547.13 cd/m^2^, an external quantum efficiency of 15.79%, and an ultra-fast steady state EL response speed of 700 ns under long pulse voltage. Moreover, we also show our AM-PeLEDs display with smooth image switching. Our work represents the most advanced performance of ultra-high-resolution perovskite quantum dot light-emitting diodes reported so far. The ultra-fast response speed combined with high resolution and high efficiency of the PeLEDs demonstrates its great potential in future applications such as high-resolution and high-refresh-rate display.

## Materials and methods

### Materials

Bromide lead (99.999%), cesium carbonate (99.9%), octanoic acid (OTAC) (99%), formamidine acetate (99%), N, N-Dimethyl dodecyl amine bromide (DDAB) (98%), N, N, N–Trioctylamine bromide (TOAB) (98%), and 1-Butyltrimethylimidazolium trifluoromethanesulfonic acid ([BMIM]OTF) (97%) were purchased from Aladdin. Toluene, chlorobenzene, acetone, ethanol, and ethyl acetate were all analytical pure and could be used directly without further purification. PEDOT: PSS (poly(3,4-ethylenedioxythiophene): poly(4-styrenesulfonicacid)), PTAA (poly[(2,4,6-trimethylphenyl) (4-phenylphenyl) amine]), lithium fluoride, and TPBI (1,3,5-tri(1-phenyl-1H-benzotriazole-2-yl) benzene) were purchased from Xi’an Polymer Light Technology Corp.

### Synthesis and purification of QDs

Preparation of precursor Solution: 0.8 mmol Cs_2_CO_3_ (0.26066 g) and 8 ml OTAC were added into a 10 ml vial, and stirred for half an hour to prepare the cesium precursor. The precursor for the dopant cationic species was prepared by mixing 0.4 mmol FA (Ac) (0.04164 g) and dissolving it in 2 ml OTAC. 1 mmol PbBr_2_ (0.367 g) and 2 mmol TOAB (1.09358 g) were dissolved in 10 ml toluene, and stirred overnight to ensure complete dissolution of PbBr_2_. For blue light synthesis, 0.6 mmol PbBr_2_ and 0.4 mmol PbCl_2_ were used instead of 1 mmol PbBr_2_, with the other conditions remaining the same.

Synthesis of FA_0.15_CS_0.85_PbBr_3_ Quantum Dots: To simplify the process and obtain quantum dots with excellent performance, the ligand-assisted reprecipitation (LARP) method developed by Zeng was adopted with some modifications^66^. For the synthesis of QDs with different ion liquid modifications, 9 ml of PbBr_2_ precursor solution was added to each vial, followed by the addition of 0 ml, 0.05 ml, 0.15 ml, and 0.25 ml and 0.35 ml ion liquid chlorobenzene solution (The increase in addition corresponds to Control, [BMIM]OTF-1, [BMIM]OTF-2, [BMIM]OTF-3, [BMIM]OTF-4. [BMIM]OTF, which were dissolved in chlorobenzene at a concentration of 5 mg/ml). Under high-speed stirring, 0.85 ml cesium source and 0.15 ml formamidine acetate precursor were quickly added, resulting in the formation of bright green color indicating the crystallization of quantum dots. After stirring for 5 minutes, 3 ml DDAB toluene solution (10 mg/ml DDAB toluene solution) was added as a long-chain dispersing ligand. The solution was stirred for another 2 minutes to ensure that DDAB was fully adsorbed on the surface of the quantum dots.

Purification: The crude quantum dot solution (13 ml) was divided equally into two centrifuge tubes, and each tube was mixed with double the volume of ethyl acetate. After centrifuging at 12000 rpm for 5 minutes, the supernatant was removed and replaced with 1.5 ml n-octane solution. The tubes were then centrifuged at 9000 rpm for another 5 minutes to remove large particles, and the quantum dot solution was removed and stored in the refrigerator for subsequent device fabrication and characterization.

### Device fabrication

Preparation of PeLEDs: Firstly, the indium tin oxide (ITO) substrate was ultrasonically cleaned with acetone, ethanol, and ultrapure water for 20 minutes and then dried in an oven. Subsequently, the filtered PEDOT: PSS 4083 solution (using a 0.22 μm water filter) was spin-coated onto the ITO substrate at 4000 rpm for 60 seconds. Then, the film was annealed at 140 °C for 15 minutes. After that, PTAA (chlorobenzene, 5 mg/mL) was spin-coated onto the PEDOT: PSS layer at 2000 rpm for 60 seconds and then annealed at 120 °C for 15 minutes. Subsequently, the substrate was transferred to a nitrogen glovebox, and the quantum dots were diluted to a concentration of 20 mg/ml. The QD layer was spin-coated onto the PTAA layer at 2000 rpm for 60 seconds and then annealed at 60 °C for 10 minutes. For devices with lithium bromide interfacial modification, a 2 nm thick lithium bromide layer was evaporated onto the PTAA layer before spin-coating the quantum dots. TPBI (35 nm), LiF (1 nm), and Al (100 nm) were deposited using a thermal evaporation system under a high vacuum of 6×10^−4^ Pa, with a shadow mask. The emission area of the device was 4 mm^2^, determined by the overlap between the ITO anode and the Al cathode.

Preparation of high-resolution PeLEDs: Firstly, preparation of patterned PMMA film, plasma the glass substrate and PDMS stamp for 10 minutes, then spin coat the prepared HPC (50 mg/ml) aqueous solution onto the glass substrate at 3000 rpm, cover the PDMS stamp on the HPC and apply uniform pressure under heating conditions. After 2 minutes, peel off the PDMS from the HPC. After the HPC is pressed into a cylindrical shape, PMMA (10 mg/ml, toluene solution) is spin-coated twice continuously at 1200 rpm, and then the entire substrate is placed on a heating table to dry the PMMA film. After drying the PMMA film, immerse the glass substrate in water. When HPC dissolves, the patterned PMMA film will detach from the glass substrate. Then, use a clean ITO substrate to bond the PMMA and remove the water surface. Heat the ITO on a heating table to firmly attach the PMMA film to the ITO. Deposition of functional layers: Referring to the preparation process of the prototype device mentioned above, various functional layers are sequentially deposited on patterned PMMA films.

Preparation of AM-PeLEDs Display: The active-matrix TFT substrate, purchased from Suzhou Ling Zhi Technology Co., Ltd. (China), features an active area of 17.28 × 17.28 mm^2^ and a pixel resolution of 64 × 64 μm. Functional layers (PEDOT: PSS, PTAA) and QD solutions were spin-coated onto the substrate based on predetermined parameters. The non-active area was cleaned (wipe off the material spin coated onto the electrode with clean acetone) prior to depositing TPBI, LiF and Al. After the device is completed, the active area is encapsulated in a glove box using UV glue and cover glass. Then connect the device to the driver box and perform display testing using an APP (both driver hardware and software are developed and provided by Suzhou Ling Zhi Technology Co., Ltd.).

### Characterizations

The ultraviolet-visible spectrophotometer (Shimadzu, UV-3600) was used to measure the absorption spectrum of different QDs. Transmission electron microscopy (TEM, Tecnai G2F20 S-TWIN) was used for observation of morphology of QDs, and nano measure software was used to measure the size distribution of QD. Fluorescence spectrophotometer (Hitachi F 4600) was utilized to collect PL spectra. Specifically, the samples were prepared by dispersing 10 μl QD ink in 4 ml octane for absorption and PL measurements. The decay lifetimes of QD modified with different ionic liquids were measured using a fluorescence lifetime measurement system (HORIBA Scientific) through time-resolved photoluminescence (TRPL). XRD samples were prepared by dropping QD ink onto a silicon wafer and tested using an X’Pert PRO diffractometer (PANalytical). X-ray photoelectron spectroscopy (XPS, Thermo Fisher Scientific, Skarab-250) was used to verify the surface chemical state of QD samples, which were prepared by spin coating QD ink onto a silicon substrate. The impedance and capacitance characteristics of the device were tested using E4990A impedance analyzer and 4200 semiconductor testing analyzer, respectively. UV1200 was used to test the energy level of the material. Atomic force microscopy (AFM, Bruker Multimode 8) and field scanning electron microscopy (SEM, Nova Nano-SEM) were employed to characterize the morphology of QD thin films. The testing of steady-state EL is based on a self-built experimental platform, using voltage mode to drive the PeLEDs to light-emitting. All TREL tests related to response speed are steady-state EL tests (based on long pulse voltage, 0.5 KHz to 15 KHz, voltage range 3 V to 7 V), and comparisons between control devices and improved devices are based on the same voltage and pulse frequency (5 V, 10 KHz). EL spectra, J-V-L characteristics, and EQE were collected using a Keithley 2400 light source, fiber optic integrating sphere, and PMA-12 spectrograph, respectively, in a nitrogen-filled glove box at room temperature for light output measurements.

## Supplementary information

Supporting Information

Supplementary video 1

Supplementary video 2

Supplementary video 3

## Data Availability

Relevant data supporting this study's key findings are available in the article and the Supplementary Information file. All raw data generated in this study are available from the corresponding author upon reasonable request.

## References

[1] Levchuk, I. et al. Brightly luminescent and color-tunable formamidinium lead halide perovskite FAPbX_3_ (X = Cl, Br, I) colloidal nanocrystals. *Nano Lett.* **17**, 2765–2770 (2017). 28388067 10.1021/acs.nanolett.6b04781

[2] Bao, C. X. et al. A multifunctional display based on photo-responsive perovskite light-emitting diodes. *Nat. Electron.* **7**, 375–382 (2024).

[3] Elkhouly, K. et al. Electrically assisted amplified spontaneous emission in perovskite light-emitting diodes. *Nat. Photonics* **18**, 132–138 (2024).

[4] Zhang, X. L. et al. Conductive colloidal perovskite quantum dot inks towards fast printing of solar cells. *Nat. Energy* **9**, 1378–1387 (2024).

[5] Wang, J. D. et al. Matched electron-transport materials enabling efficient and stable perovskite quantum-dot-based light-emitting diodes. *Angew. Chem. Int. Ed.* **63**, e202410689 (2024). 10.1002/anie.202410689 39072910

[6] Lee, K. et al. Highly efficient pure red light-emitting diodes through surface bromination of CsPbI_3_ perovskite nanocrystals for skin-attachable displays. *Mater. Today* **75**, 2–10 (2024).

[7] Li, Y. M. et al. Stable and efficient CsPbI_3_ quantum-dot light-emitting diodes with strong quantum confinement. *Nat. Commun.* **15**, 5696 (2024). 38972890 10.1038/s41467-024-50022-8 PMC11228028

[8] Zhang, J. F. et al. Fine-tuning crystal structures of lead bromide perovskite nanocrystals through trace cadmium(II) doping for efficient color-saturated green LEDs. *Angew. Chem. Int. Ed.* **63**, e202403996 (2024). 10.1002/anie.202403996 38679568

[9] Li, H. J. et al. Thermal management towards ultra-bright and stable perovskite nanocrystal-based pure red light-emitting diodes. *Nat. Commun.* **15**, 6561 (2024). 39095426 10.1038/s41467-024-50634-0 PMC11297279

[10] Kim, D. H. et al. Surface-binding molecular multipods strengthen the halide perovskite lattice and boost luminescence. *Nat. Commun.* **15**, 6245 (2024). 39048540 10.1038/s41467-024-49751-7 PMC11269598

[11] Luo, C. Z. et al. Ultrahigh-resolution, high-fidelity quantum dot pixels patterned by dielectric electrophoretic deposition. *Light Sci. Appl.* **13**, 273 (2024). 39327426 10.1038/s41377-024-01601-3 PMC11427692

[12] Lee, H. E. et al. Micro light-emitting diodes for display and flexible biomedical applications. *Adv. Funct. Mater.* **29**, 1808075 (2019).

[13] Liu, D. et al. Nondestructive direct optical patterning of perovskite nanocrystals with carbene-based ligand cross-linkers. *ACS Nano* **18**, 6896–6907 (2024). 38376996 10.1021/acsnano.3c07975

[14] Bai, W. H. et al. Microscale perovskite quantum dot light-emitting diodes (Micro-PeLEDs) for full-color displays. *Adv. Opt. Mater.* **10**, 2200087 (2022).

[15] Cho, H. et al. Direct optical patterning of quantum dot light-emitting diodes via in situ ligand exchange. *Adv. Mater.* **32**, 2003805 (2020). 10.1002/adma.202003805 33002295

[16] Liu, D. et al. Direct optical patterning of perovskite nanocrystals with ligand cross-linkers. *Sci. Adv.* **8**, abm8433 (2022). 10.1126/sciadv.abm8433 PMC8926341 35294230

[17] Li, D. Y. et al. Efficient red perovskite quantum dot light-emitting diode fabricated by inkjet printing. *Mater. Futures* **1**, 015301 (2022).

[18] Wei, C. T. et al. A universal ternary-solvent-ink strategy toward efficient inkjet-printed perovskite quantum dot light-emitting diodes. *Adv. Mater.* **34**, 2107798 (2022). 10.1002/adma.202107798 34990514

[19] Kwon, J. I. et al. Ultrahigh-resolution full-color perovskite nanocrystal patterning for ultrathin skin-attachable displays. *Sci. Adv.* **8**, eadd0697 (2022). 36288304 10.1126/sciadv.add0697 PMC9604611

[20] Huang, Y. G. et al. Mini-LED, Micro-LED and OLED displays: present status and future perspectives. *Light Sci. Appl.* **9**, 105 (2020). 32577221 10.1038/s41377-020-0341-9 PMC7303200

[21] Shan, Q. S. et al. Perovskite light-emitting/detecting bifunctional fibres for wearable LiFi communication. *Light Sci. Appl.* **9**, 163 (2020). 33014358 10.1038/s41377-020-00402-8 PMC7494868

[22] Yonebayashi, R. et al. High refresh rate and low power consumption AMOLED panel using top-gate n-oxide and p-LTPS TFTs. *J. Soc. Inf. Disp.* **28**, 350–359 (2020).

[23] Hsiang, E. L. et al. Prospects and challenges of mini-LED, OLED, and micro-LED displays. *J. Soc. Inf. Disp.* **29**, 446–465 (2021).

[24] Elkhouly, K. et al. Perovskite light emitting diode characteristics: the effects of electroluminescence transient and hysteresis. *Adv. Opt. Mater.* **8**, 2000941 (2020).

[25] Kumawat, N. K., Tress, W. & Gao, F. Mobile ions determine the luminescence yield of perovskite light-emitting diodes under pulsed operation. *Nat. Commun.* **12**, 4899 (2021). 34385427 10.1038/s41467-021-25016-5 PMC8361013

[26] Tsai, C. L. et al. Bright and fast-response perovskite light-emitting diodes with an ICBA:modified-C_60_ nanocomposite electrical confinement layer. *Nanoscale* **12**, 4061–4068 (2020). 32022049 10.1039/c9nr10246a

[27] Xu, M. M. et al. A transient-electroluminescence study on perovskite light-emitting diodes. *Appl. Phys. Lett.* **115**, 041102 (2019).

[28] Bao, C. X. et al. Bidirectional optical signal transmission between two identical devices using perovskite diodes. *Nat. Electron.* **3**, 156–164 (2020). 32226921 10.1038/s41928-020-0382-3 PMC7100905

[29] Huang, Y. C., Xu, D. X. & Chen, S. M. P-9.1: investigation of electroluminescent response of quantum dot light-emitting diodes. *SID Symp. Dig. Tech. Pap.* **49**, 656–659 (2018).

[30] Blauth, C., Mulvaney, P. & Hirai, T. Transient overshoot and storage of charge carriers on ligands in quantum dot LEDs. *J. Appl. Phys.* **126**, 075501 (2019).

[31] Zhao, L. F. et al. Nanosecond-pulsed perovskite light-emitting diodes at high current density. *Adv. Mater.* **33**, 2104867 (2021). 10.1002/adma.202104867 34477263

[32] Shen, C. et al. High performance and stable pure-blue quasi-2D perovskite light-emitting diodes by multifunctional zwitterionic passivation engineering. *Adv. Photonics* **6**, 026002 (2024).

[33] Li, N. et al. Electroluminescence and photo-response of inorganic halide perovskite bi-functional diodes. *Nanophotonics* **7**, 1981–1988 (2018).

[34] Qu, W. et al. Preferred orientation and phase distribution of quasi-2D perovskite for bifunctional light-emitting photodetectors. *Nano Lett.* **24**, 7593–7600 (2024). 38869928 10.1021/acs.nanolett.4c00927

[35] Li, Z. C. et al. Charge injection engineering at organic/inorganic heterointerfaces for high-efficiency and fast-response perovskite light-emitting diodes. *Nat. Commun.* **14**, 6441 (2023). 37833266 10.1038/s41467-023-41929-9 PMC10575909

[36] Gao, Y. et al. Microsecond-response perovskite light-emitting diodes for active-matrix displays. *Nat. Electron.* **7**, 487–496 (2024).

[37] Zeng, Q. G. et al. Ionic liquid-induced in situ deposition of perovskite quantum dot films with a photoluminescence quantum yield of over 85%. *Nanoscale* **13**, 20067–20077 (2021). 34846058 10.1039/d1nr05528c

[38] Hoang, M. T. et al. A facile, environmentally friendly synthesis of strong photo-emissive methylammonium lead bromide perovskite nanocrystals enabled by ionic liquids. *Green. Chem.* **22**, 3433–3440 (2020).

[39] Luo, Y. et al. A multifunctional ionic liquid additive enabling stable and efficient perovskite light-emitting diodes. *Small* **18**, 2200498 (2022). 10.1002/smll.202200498 35419974

[40] Niu, T. T. et al. Ionic liquids-enabled efficient and stable perovskite photovoltaics: progress and challenges. *ACS Energy Lett.* **6**, 1453–1479 (2021).

[41] Yu, W. J. et al. Separating crystal growth from nucleation enables the in situ controllable synthesis of nanocrystals for efficient perovskite light-emitting diodes. *Adv. Mater.* **35**, 2301114 (2023). 10.1002/adma.202301114 37314026

[42] Li, J. C. et al. Electronic coordination effect of the regulator on perovskite crystal growth and its high-performance solar cells. *ACS Appl. Mater. Interfaces* **12**, 19439–19446 (2020). 32252516 10.1021/acsami.0c00762

[43] Yang, K. Y. et al. Interface engineering with ionic liquid for achieving efficient Quasi-2D perovskite light-emitting diodes. *Chem. Eng. J.* **483**, 149291 (2024).

[44] Zhou, X. et al. Doping amino-functionalized ionic liquid in perovskite crystal for enhancing performances of hole-conductor free solar cells with carbon electrode. *Chem. Eng. J.* **372**, 46–52 (2019).

[45] Yang, J. N. et al. High color purity and efficient green light-emitting diode using perovskite nanocrystals with the size overly exceeding bohr exciton diameter. *J. Am. Chem. Soc.* **143**, 19928–19937 (2021). 34766754 10.1021/jacs.1c09948

[46] Zhou, Q. S. et al. Multifunctional chemical bridge and defect passivation for highly efficient inverted perovskite solar cells. *ACS Energy Lett.* **6**, 1596–1606 (2021).

[47] Mei, X. Y. et al. Stabilizing dynamic surface of highly luminescent perovskite quantum dots for light-emitting diodes. *Chem. Eng. J.* **453**, 139909 (2023).

[48] Yang, J. N. et al. Potassium bromide surface passivation on CsPbI_3-*x*_Br_*x*_ nanocrystals for efficient and stable pure red perovskite light-emitting diodes. *J. Am. Chem. Soc.* **142**, 2956–2967 (2020). 31902206 10.1021/jacs.9b11719

[49] Zhang, J. B. et al. Highly luminescent and stable CsPbI_3_ perovskite nanocrystals with sodium dodecyl sulfate ligand passivation for red-light-emitting diodes. *J. Phys. Chem. Lett.* **12**, 2437–2443 (2021). 33661637 10.1021/acs.jpclett.1c00008

[50] Fang, T. et al. Sulfonate additive simultaneously suppresses interstitials and vacancies toward efficient and stable perovskite quantum dot LEDs. *Adv. Opt. Mater.* **12**, 2302253 (2023).

[51] Liu, Y. et al. Ligands for CsPbBr_3_ perovskite quantum dots: the stronger the better?. *Chem. Eng. J.* **453**, 139904 (2023).

[52] Yang, D. D. et al. CsPbBr_3_ quantum dots 2.0: benzenesulfonic acid equivalent ligand awakens complete purification. *Adv. Mater.* **31**, 1900767 (2019). 10.1002/adma.201900767 31172615

[53] Liu, Y. F. et al. Highly soluble CsPbBr_3_ perovskite quantum dots for solution-processed light-emission devices. *ACS Appl. Nano Mater.* **4**, 1162–1174 (2021).

[54] Ye, Y. L. et al. Ultra-low EQE roll-off and marvelous efficiency perovskite quantum-dots light-emitting-diodes achieved by ligand passivation. *Nano Energy* **90**, 106583 (2021).

[55] Saidaminov, M. I. et al. Inorganic lead halide perovskite single crystals: phase-selective low-temperature growth, carrier transport properties, and self-powered photodetection. *Adv. Opt. Mater.* **5**, 1600704 (2016).

[56] Chen, Z. M. et al. Recombination dynamics study on nanostructured perovskite light-emitting devices. *Adv. Mater.* **30**, 1801370 (2018). 10.1002/adma.201801370 30088297

[57] Xiao, X. T. et al. Capacitance–voltage characteristics of perovskite light-emitting diodes: Modeling and implementing on the analysis of carrier behaviors. *Appl. Phys. Lett.* **120**, 243501 (2022).

[58] Lv, S. H. et al. Operating mechanism of quantum-dot light-emitting diodes under alternating current-drive. *IEEE Electron Device Lett.* **43**, 256–259 (2022).

[59] Yang, K. Y. et al. Interface-induced crystallinity enhancement of perovskite quantum dots for highly efficient light-emitting diodes. *ACS Appl. Mater. Interfaces* **15**, 40062–40069 (2023). 37552832 10.1021/acsami.3c07302

[60] Li, X. S. et al. A multifunctional small-molecule hole-transporting material enables perovskite QLEDs with EQE exceeding 20. *ACS Energy Lett.* **8**, 1445–1454 (2023).

[61] Fang, T. et al. Perovskite QLED with an external quantum efficiency of over 21% by modulating electronic transport. *Sci. Bull.* **66**, 36–43 (2021). 10.1016/j.scib.2020.08.025 36654311

[62] Liu, M. M. et al. Suppression of temperature quenching in perovskite nanocrystals for efficient and thermally stable light-emitting diodes. *Nat. Photonics* **15**, 379–385 (2021).

[63] Zhao, H. F. et al. High-brightness perovskite light-emitting diodes based on FAPbBr_3_ nanocrystals with rationally designed aromatic ligands. *ACS Energy Lett.* **6**, 2395–2403 (2021).

[64] Xu, L. M. et al. A bilateral interfacial passivation strategy promoting efficiency and stability of perovskite quantum dot light-emitting diodes. *Nat. Commun.* **11**, 3902 (2020). 32764550 10.1038/s41467-020-17633-3 PMC7413529

[65] Mao, C. M. et al. Ultra-high-resolution perovskite quantum dot light-emitting diodes. *Adv. Opt. Mater.* **11**, 2202058 (2023).

[66] Song, J. Z. et al. Room-temperature triple-ligand surface engineering synergistically boosts ink stability, recombination dynamics, and charge injection toward EQE-11.6% perovskite QLEDs. *Adv. Mater.* **30**, 1800764 (2018). 10.1002/adma.201800764 29888521

