# Supplementary material for: Nanosecond response perovskite quantum dot light-emitting diodes with ultra-high resolution for active display application
Source: Light Sci Appl. 2025 Aug 21;14:285. doi: 10.1038/s41377-025-01959-y (PMC12370927; doi:10.1038/s41377-025-01959-y)
Supplement: Supplementary file 1 — Supporting Information [file 41377_2025_1959_MOESM1_ESM.docx]

**Supporting Information**

**Nanosecond Response Perovskite Quantum Dot Light-Emitting Diode with Ultra-High Resolution for Active Display Application**

*Qingkai Zhang^1^, Kaiyu Yang^1,2^*,* *Chengyu Luo^1^, Zhihan Lin^1^, Weiguo Chen^1^, Yongsheng Yu^1^, Hailong Hu ^1,2^, Fushan Li^1,2^**

^1^ College of Physics and Information Engineering, Fuzhou University, Fuzhou, People’s Republic of China

^2^ Fujian Science and Technology Innovation Laboratory for Optoelectronic Information of China, Fuzhou, People’s Republic of China.

E-mail addresses: [kaikaibrian@fzu.edu.cn](mailto:kaikaibrian@fzu.edu.cn) (K. Yang); fsli@fzu.edu.cn (F. Li)

**
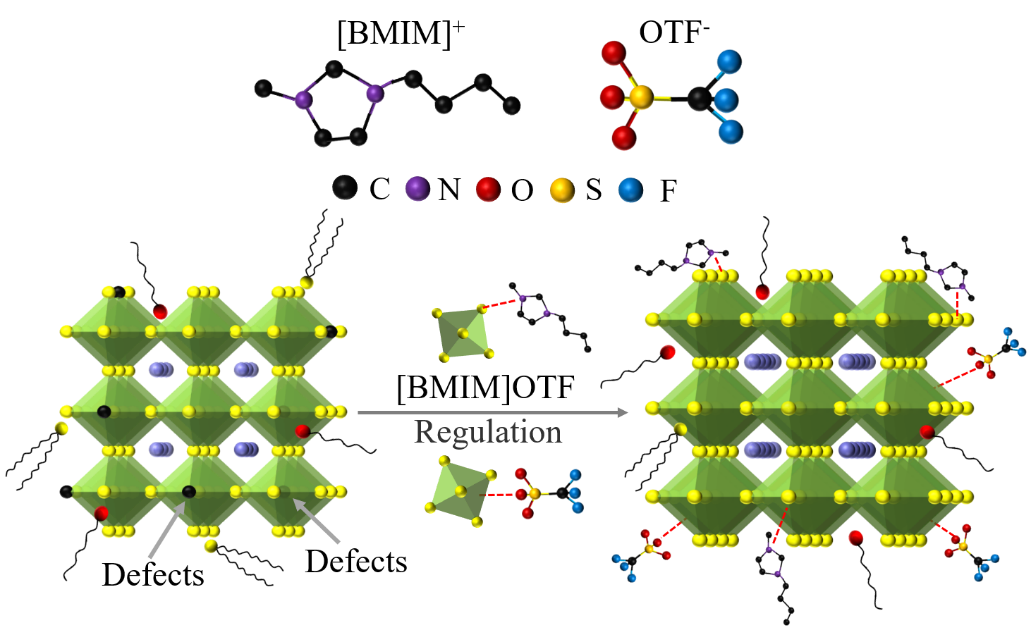
**

**Fig.S1**. The molecular structure of [BMIM]OTF and its binding on QDs surface.

(c)

(c)

(a)


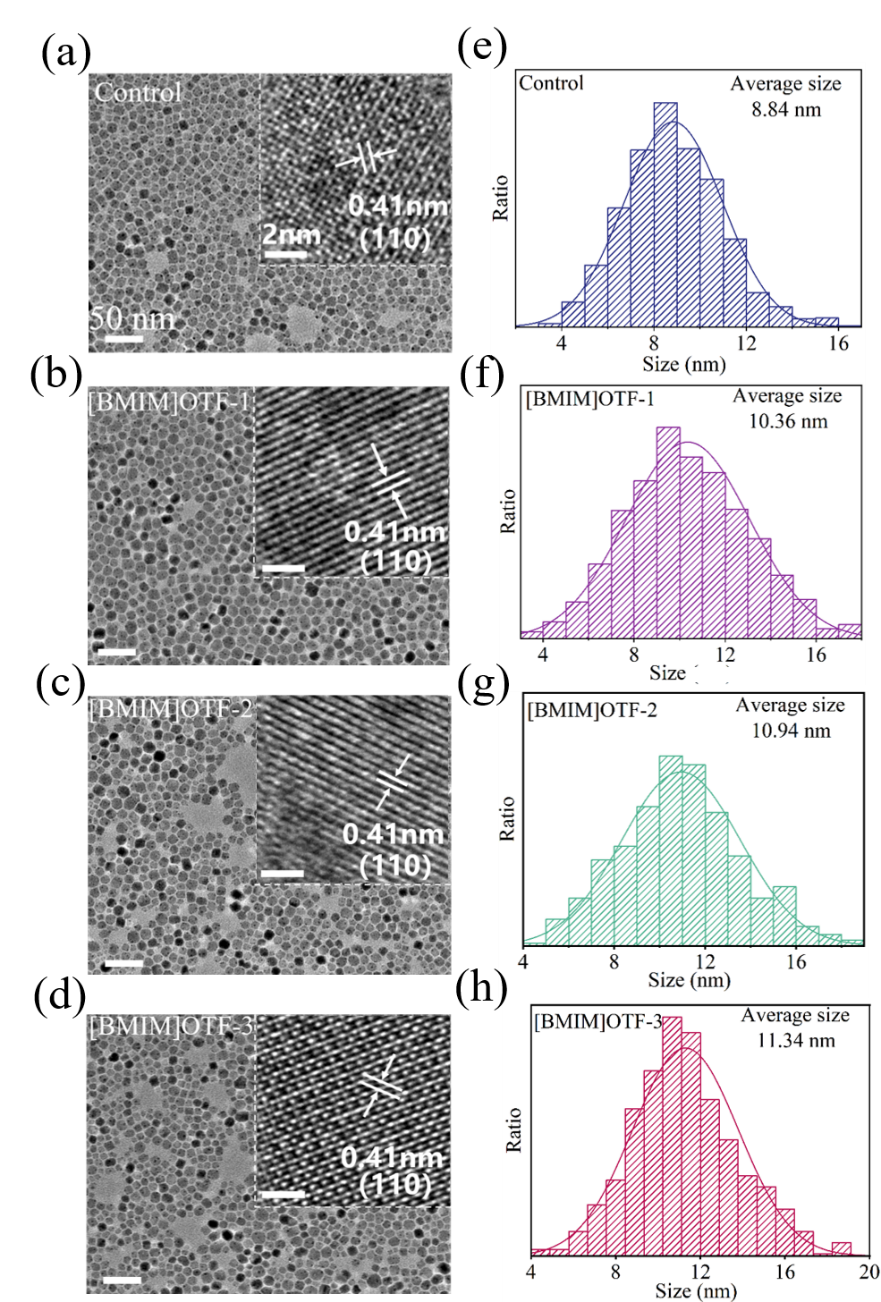


**Fig.S2**. (a-d) TEM image of QDs, illustration reveals lattice spacing. (e-h) TEM size distribution of QDs.

(c)

As shown in Fig. S1a-d, the lattice spacing is 0.41 nm, corresponding to the (110) crystal plane of the QDs. The size distribution of QDs was statistically analyzed by nano measure software. The average sizes of different QDs were 8.84, 10.36, 10.94 and 11.34 nm. This proves that the addition of [BMIM]OTF contributes to the growth of QDs.


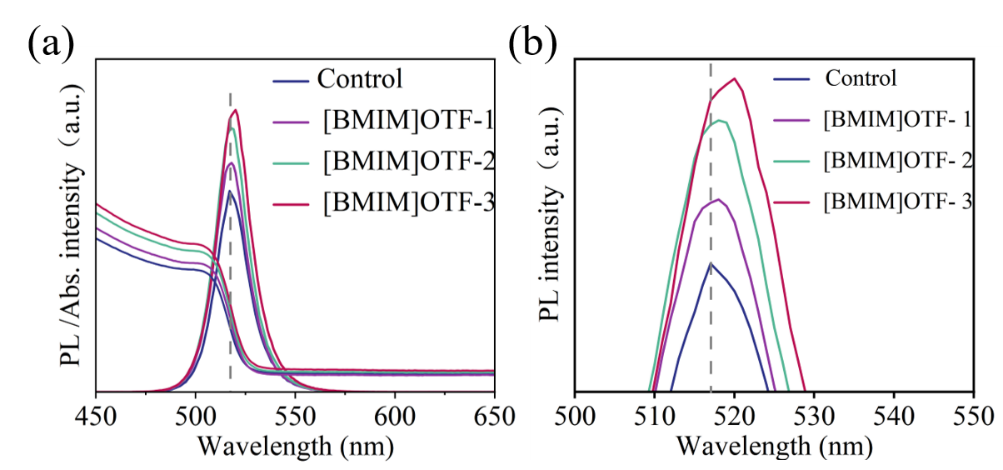


**Fig.S3.** (a-b) PL peak diagram for different QDs.

The PL peaks of QDs are located at 517 nm, 518 nm, 518 nm and 520 nm.

**
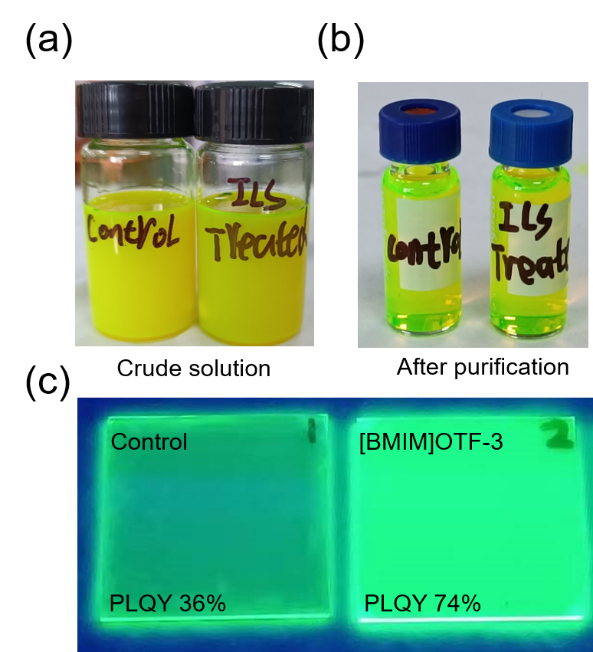
**

**Fig.S4.** (a-b) Control and [BMIM] OTF regulated synthesis solution display of QDs. (c) Films PLQY of control and [BMIM] OTF-3 QDs layer.

The thin film of [BMIM] OTF-3 QDs exhibits brighter green luminescence and higher PLQY, indicating the strong passivation effect of [BMIM]OTF for QDs.


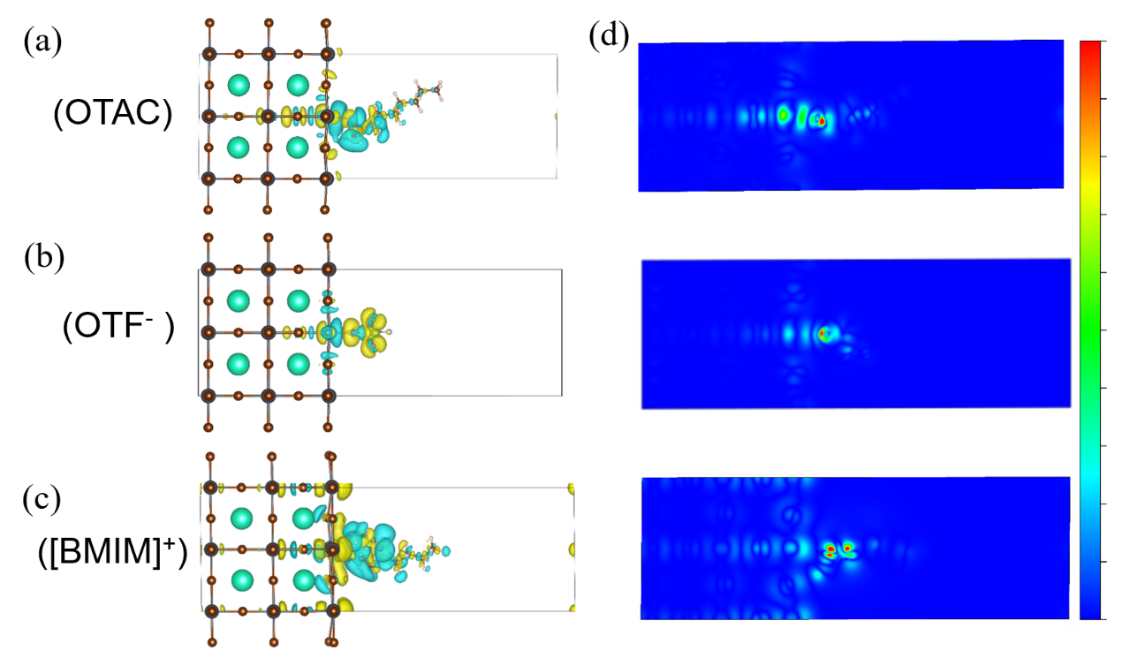


**Fig.S5.** (a-c) Three dimensional and (d) two-dimensional differential charge map after adsorption of charges.

The calculation process was as follows: the QD surface was set to have uncoordinated Pb^2+^ ions and A-site vacancies, and the binding of exposed Pb^2+^ with -COO^−^ groups (from the original ligand Octanoic acid, OTAC) and -SO_3_CF_3_^−^ (OTF^-^ ions) and the positively charged [BMIM]^+^ ion groups with surface Br^−^ ions were calculated.


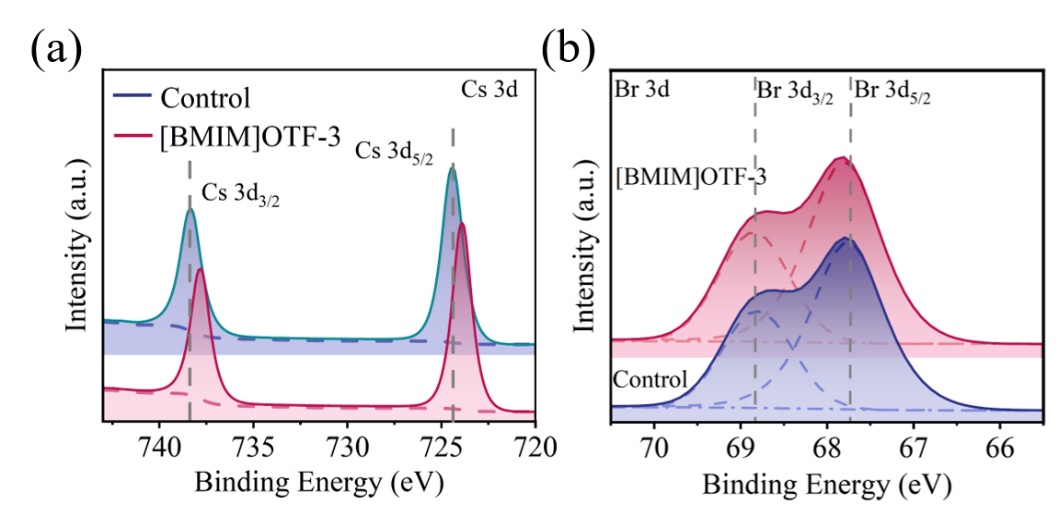


**Fig.S6.** (a) Cs-3d HRXPS spectrum of QDs. (b) Br-3d HRXPS spectrum of QDs.

In the Cs-3d spectrum, the binding energy of Cs-3d for the control QDs is at 738.3 eV and 724.4 eV, while after adding [BMIM]OTF, the Cs-3d peak shifted to 737.8 eV and 723.9 eV. The decrease in the binding energy of Cs-3d indicates a decrease in the cationic charge around the Cs^+^ ions, which can be attributed to the strong coordination between the lone electron pair of the S=O bond in [BMIM]OTF and the uncoordinated Cs^+^ cation, resulting in a reduction in the surface dangling bonds on the Cs^+^ cation which means an effective passivation of the Cs^+^ vacancy.


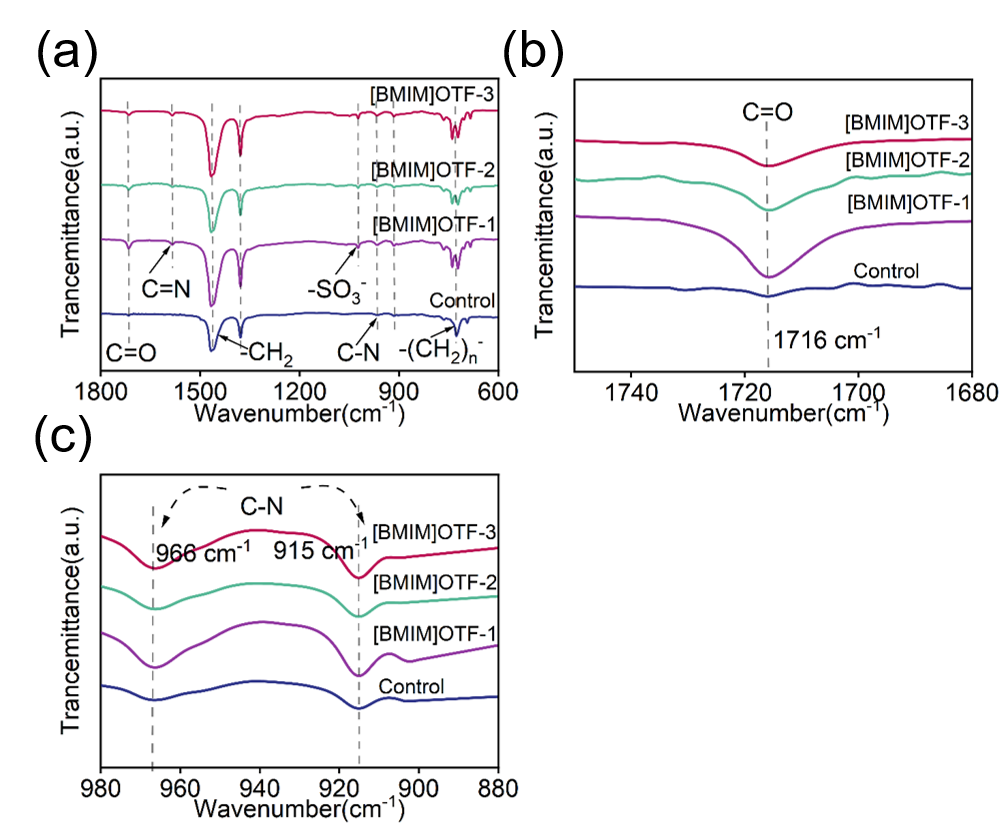


**Fig.S7.** (a) Fourier transform infrared full spectroscopy (FTIR) of QDs. (b) C=O bond vibration in the FTIR spectrum of OTAC ligands. (c) C-N bond vibration in the FTIR spectrum of DDAB ligands.


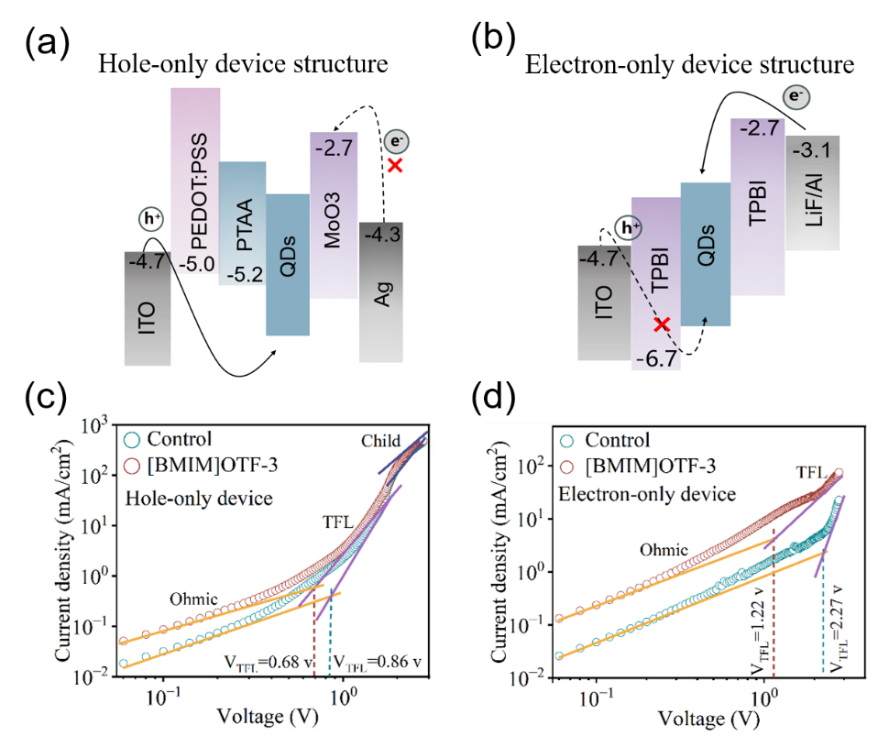


**Fig.S8.** (a-b) Device structure of Hole-only and Electron-only. (c-d) Voltage - Current density diagram of hole-only and electron-only devices.

The defect state density of hole-only and electron-only devices was calculated by the space charge limited current (SCLC) model. For hole-only device: The trap-filled limit voltage decreased from 0.86 V to 0.68 V. For electron-only device: The trap-filled limit voltage decreased from 2.27 V to 1.12 V. By formula:

N *_trap_* =2ε*_0_*ε*_r_*V*_TFL_*/qL^2^ (1)

where q is the electronic charge, L is the thickness of the QD layer (23 nm), ε*_0_* and ε*_r_* are the vacuum permittivity and average relative permittivity of CsPbBr_3_ (≈22), respectively. The trap-filled limit voltage (V*_TFL_*) of the single-carrier devices can be obtained from the J-V curves of the hole-only or electron-only devices. After the addition of [BMIM]OTF, the trap density of the hole-only device decreased from 1.96×10^18^ cm^−3^ to 1.55×10^18^ cm^−3^, and the trap density of the electron-only device decreased from 5.18×10^18^ cm^−3^ to 2.55×10^18^ cm^−3^.


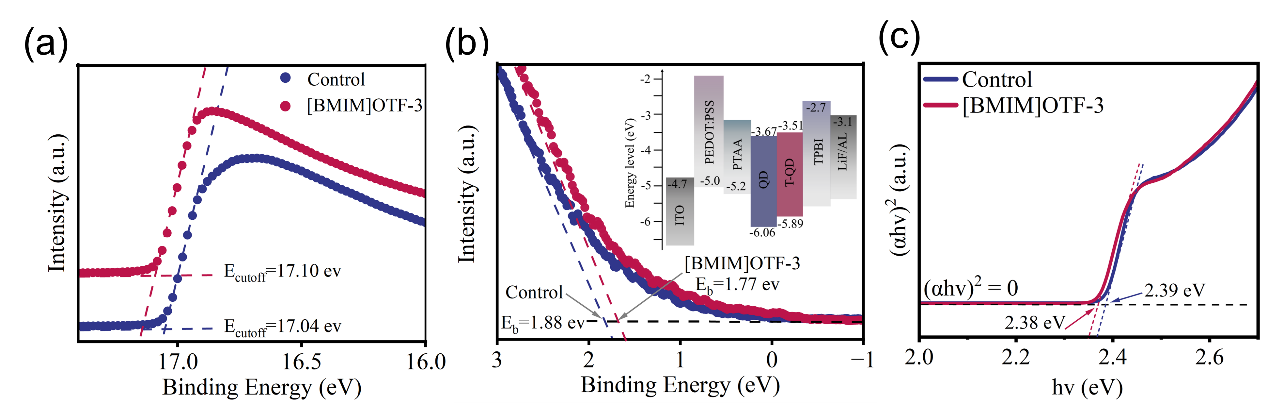


**Fig. S9.** (a) UPS secondary electron cut-off of QDs films. (b) The energy difference between the valence band and Fermi edge of QDs films, The illustration diagram of energy level structure. (c) Band gap of QDs calculated by absorption.

The work function, HOMO energy level and LUMO energy level of QDs layer was calculated by UPS. For work function:

Φ = 21.22 eV – E*_cutoff_*  (2)

There E*_cutoff_* represent secondary electron cut-off, control and [BMIM] OTF-3 QDs at 17.04 eV and 17.10 eV. Therefore, the calculated work functions are 4.18 eV and 4.12 eV, respectively. Considering that the energy difference between the valence band and Fermi edge is 1.88 eV and 1.77 eV, and the band gaps of the quantum dots are 2.39 eV and 2.38 eV, respectively (Fig. S9c), thus the valence band of the QDs layer is increased from -6.06 eV to -5.89 eV, and the corresponding conduction band increased from -3.67 eV to -3.51 eV.


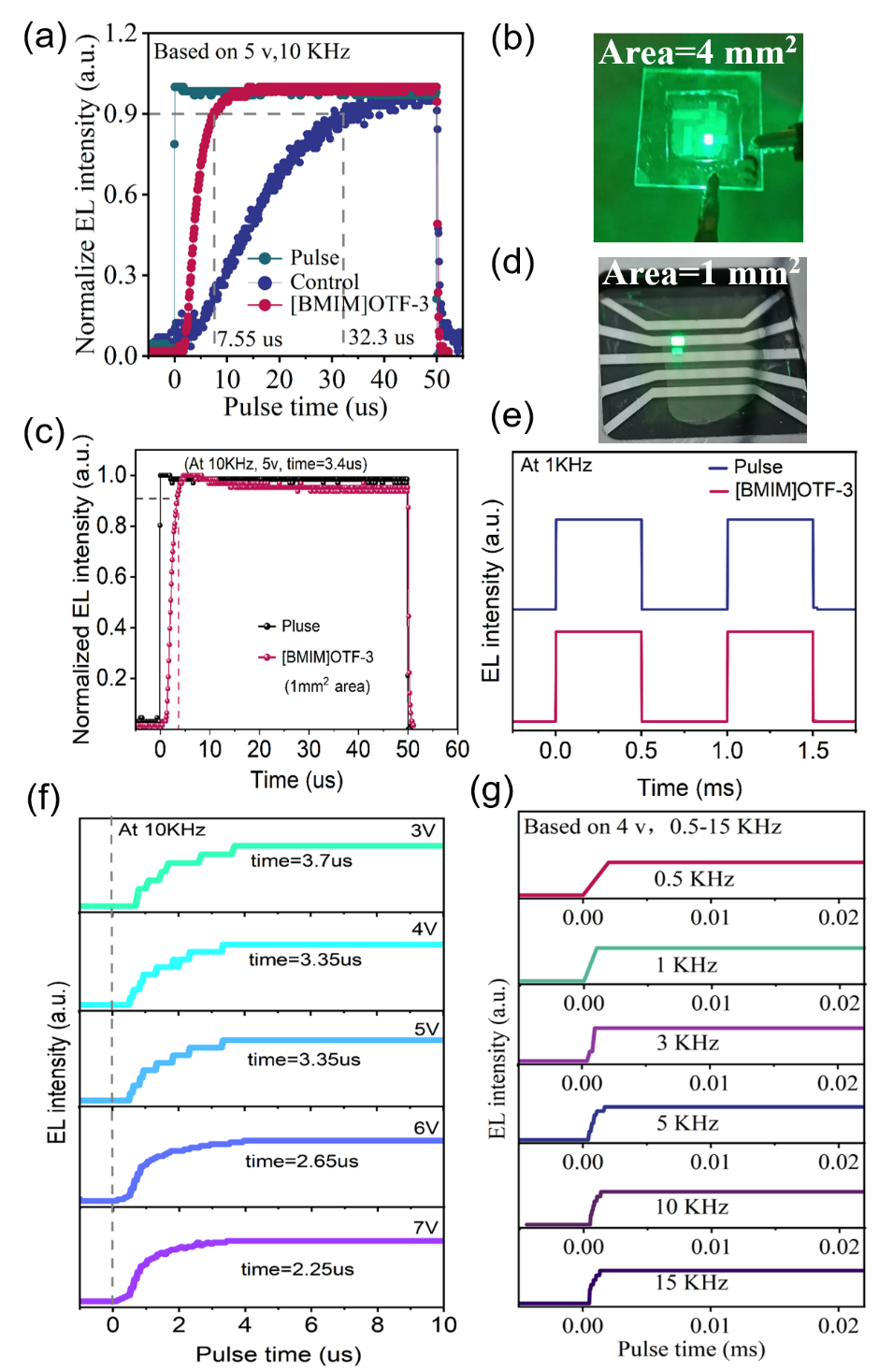


**Fig.S10.** (a) Comparison of steady-state EL Response time (TREL) for Control and [BMIM] OTF of 4 mm^2^ light-emitting area devices. (b) Physical pictures of 4 mm^2^ light-emitting area devices. (c) Steady-state EL Response of 1 mm^2^ light-emitting area. (d) Physical pictures of 1 mm^2^ light-emitting area devices. (e-f) The response speed of the front and rear pulse remains unchanged, as well as the response speed of devices with different voltages. (g) The response based on different frequencies.


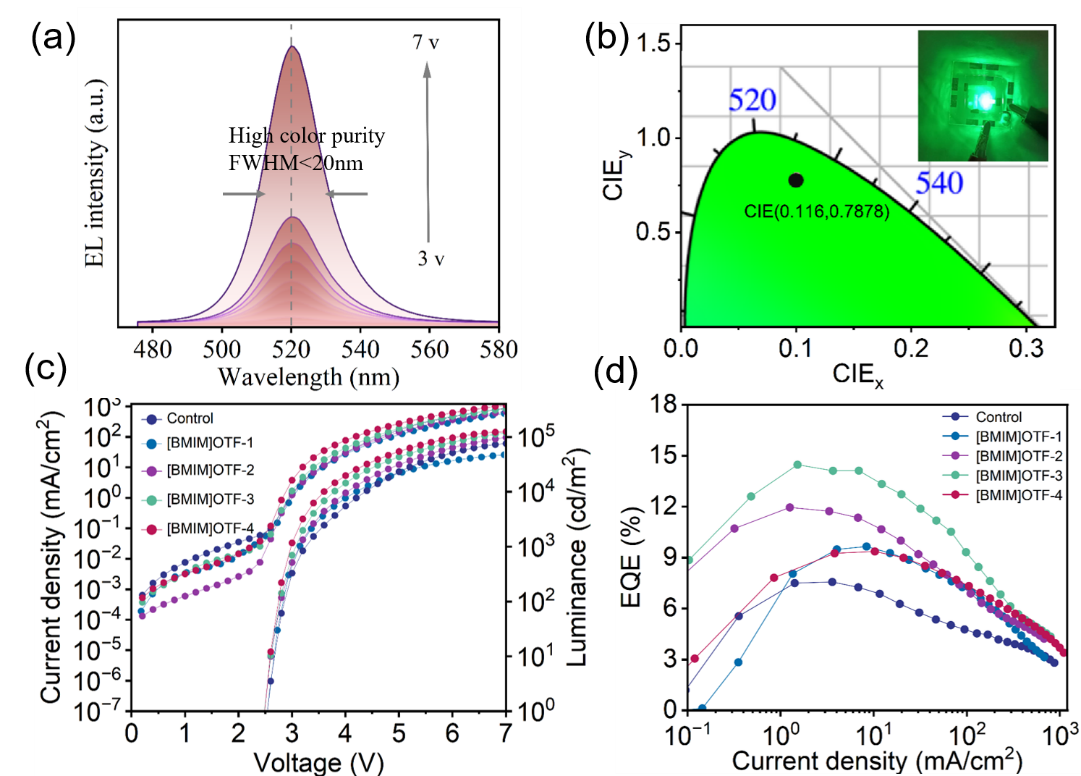


**Fig.S11.** (a) EL diagram of different voltages. (b) CIE coordinates. (c-d) Current-voltage-brightness and efficiency (EQE) variation curve of different [BMIM] OTF addition PeLEDs.

**
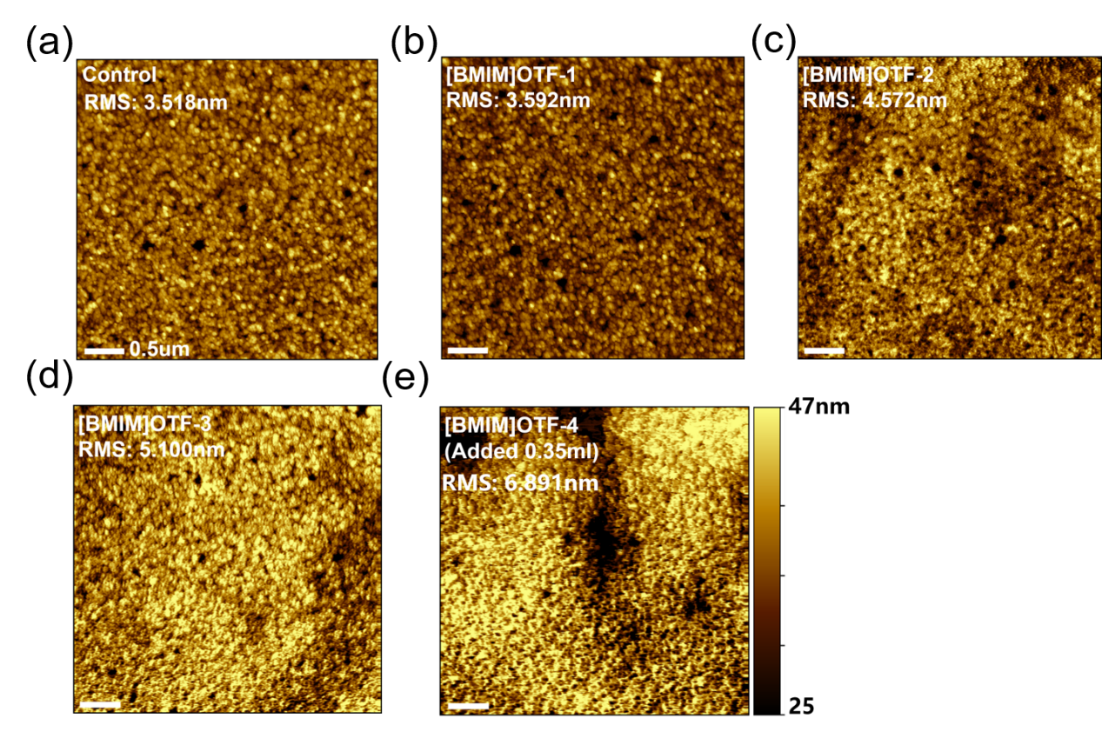
**

**Fig.S12.** AFM images of QDs layer.

With the addition of [BMIM] OTF, the surface roughness (AFM) of the QDs film increases. When excessive [BMIM] OTF-4 is added, the AFM of the film is higher (6.891 nm), which may lead to leakage current.


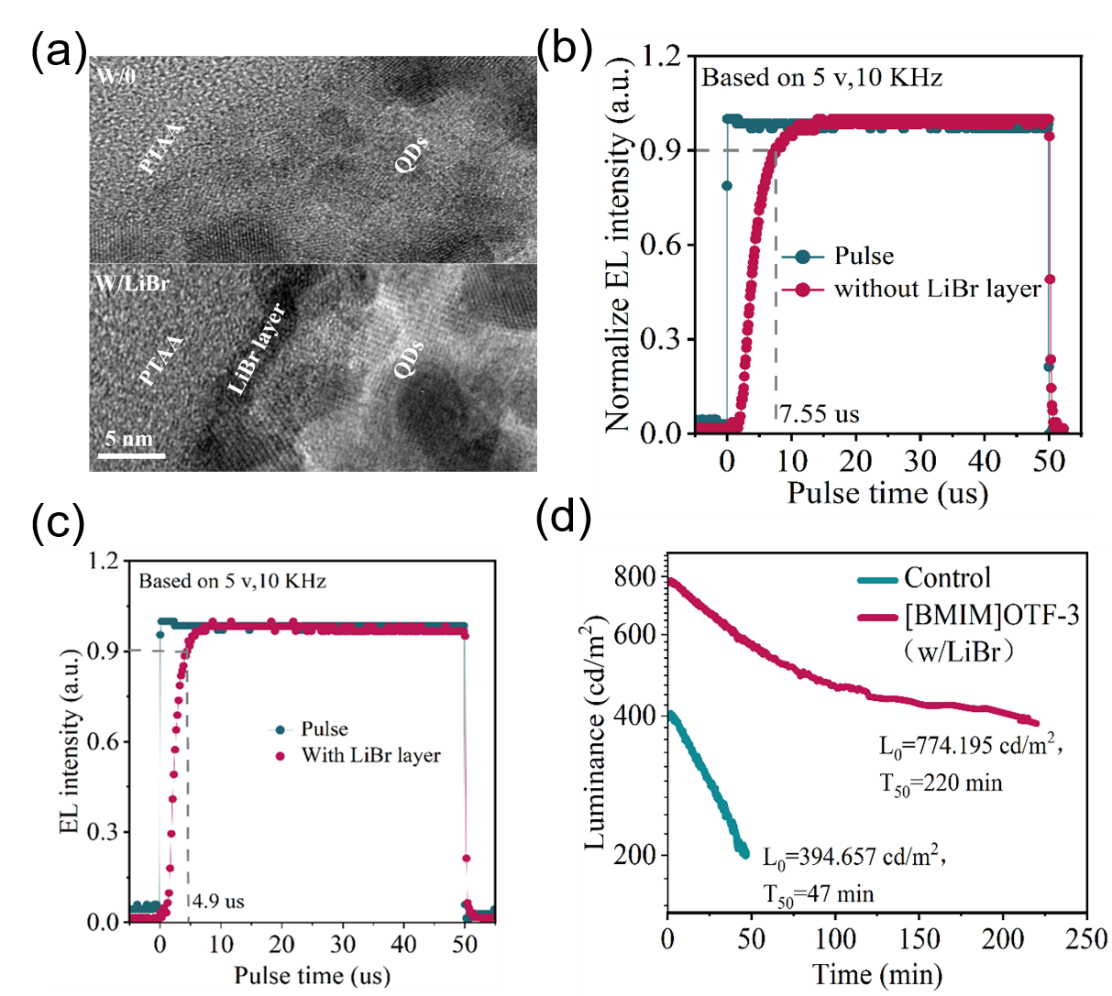


**Fig.S13.** (a) TEM images of device with and without lithium bromide interface. (b-c) Steady-state EL response time comparison of devices with and without lithium bromide interface layer. (d) T_50_ lifetime curve for control and [BMIM] OTF passivation devices.

Calculate the T_50_ lifetime (@ L_0_=100 cd m^-2^) using the formula:

(L_0_) *^n^* × T_50_ = constant (3)

The lifetime of control device with the L_0_ of 394.657 cd m^-2^ was 47 min, while the treated device exhibits a lifetime with the L_0_ of with the L_0_ of 774.195 cd m^-2^ was 220 min. Setting acceleration factor n (~ 1.75), the operational lifetimes of the control and treated were calculated to be about 8.62 h and 131.87 h at L_0_=100 cd m^-2^ respectively.


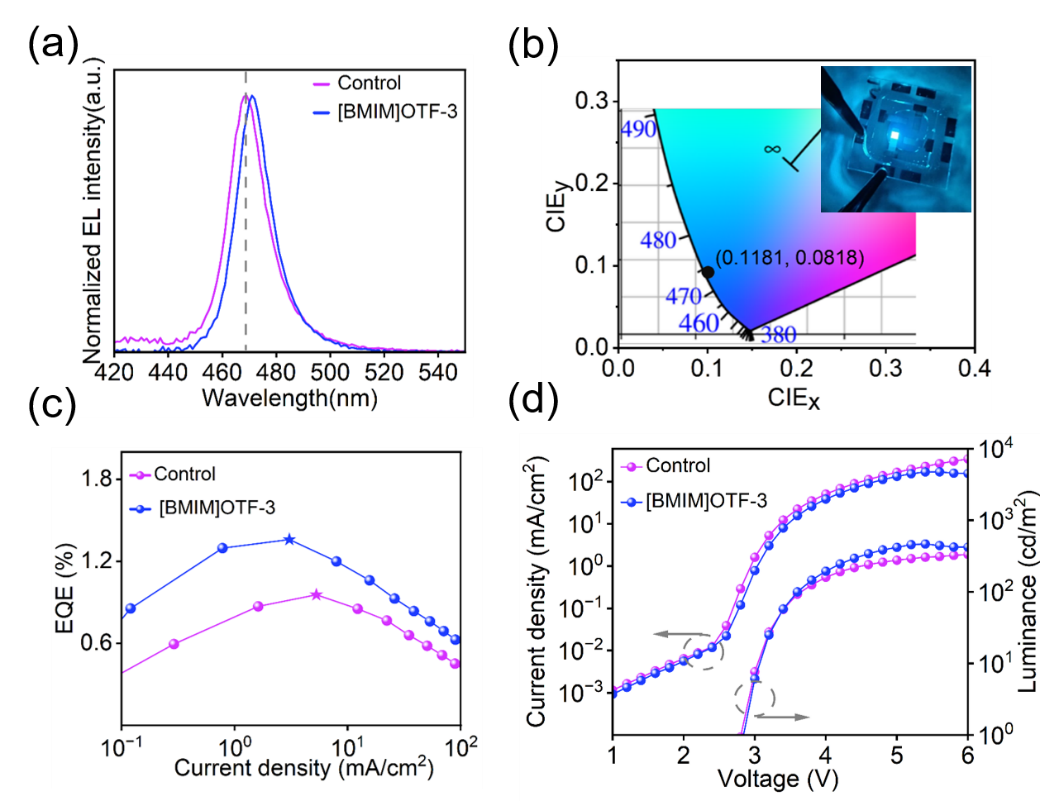


**Fig.S14.** (a) EL spectrum. (b) CIE coordinates of pure-blue PeLEDs. (c-d) Current-Voltage-Brightness, EQE curves of pure- blue PeLEDs.


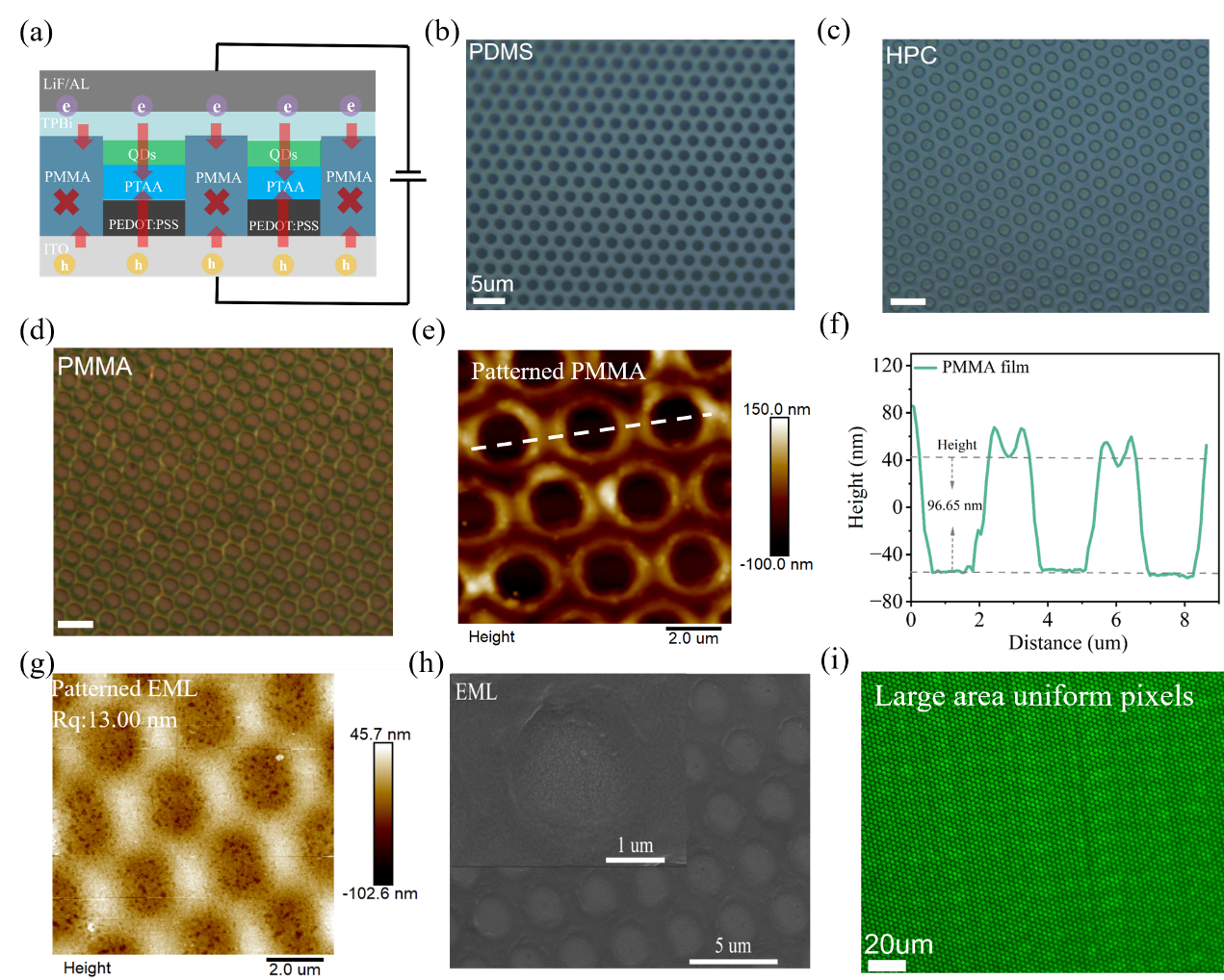


**Fig.S15.** (a) High resolution device structure diagram. (b-d) 2D microscope image of PDMS, HPC and honeycomb shaped PMMA grid. (e) AFM image of patterned PMMA template. (f) Depth map of PMMA template structure. (g) AFM image of patterned EML layer. (h) SEM image of patterned EML layer. (i) Large area optical microscope images of high-resolution QDs layers.


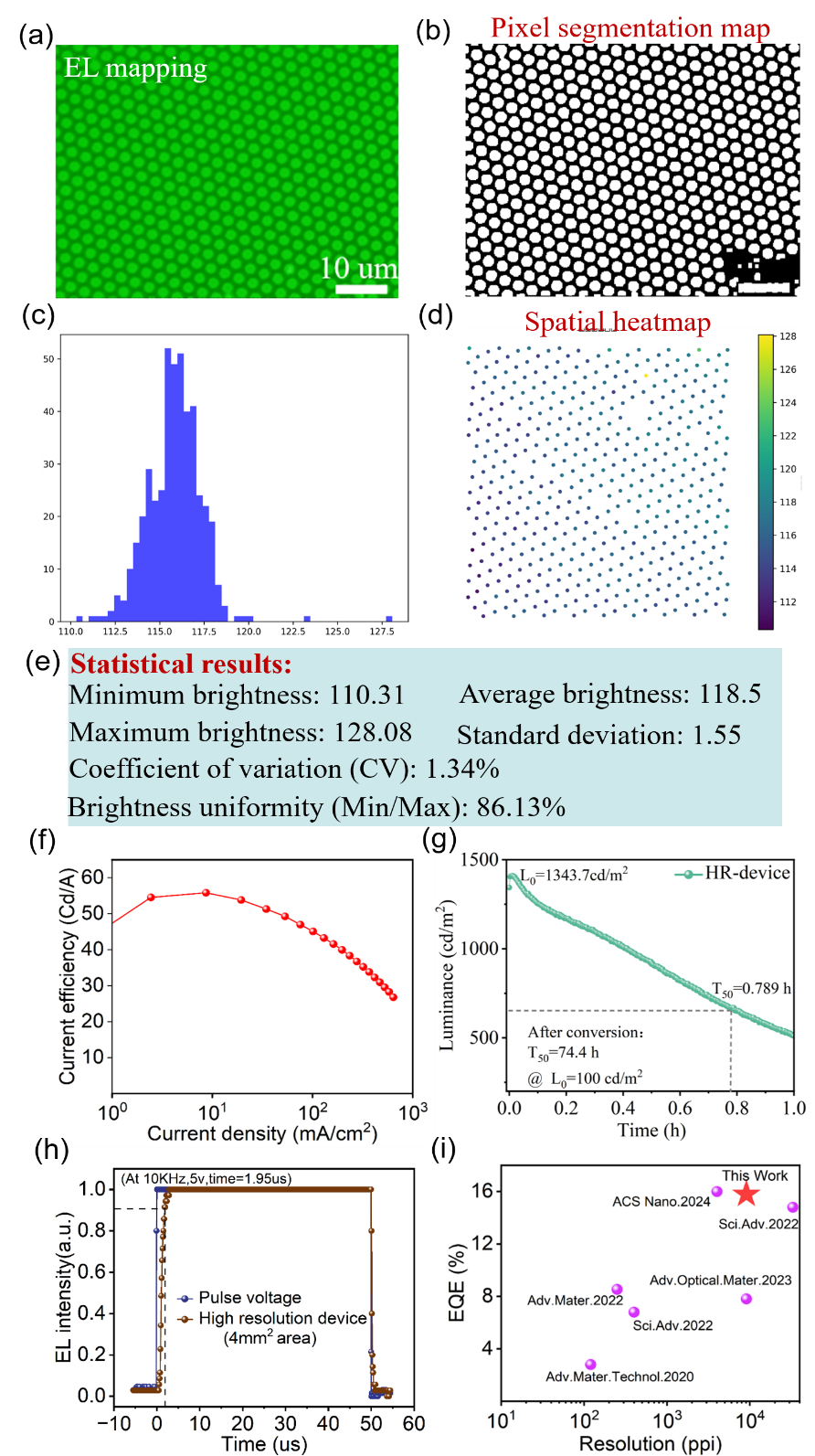


**Fig.S16.** (a) Electroluminescence microscopy images. (b) Pixel statistical segmentation images. (c) Brightness statistical histogram. (d-e) Thermal images of brightness spatial distribution and Statistical results. (f-g) The current efficiency and lifetime curve of high-resolution devices. (h) TREL plot of high-resolution devices with 4 mm^2^ light-emitting area. (i) Recent research progress chart.


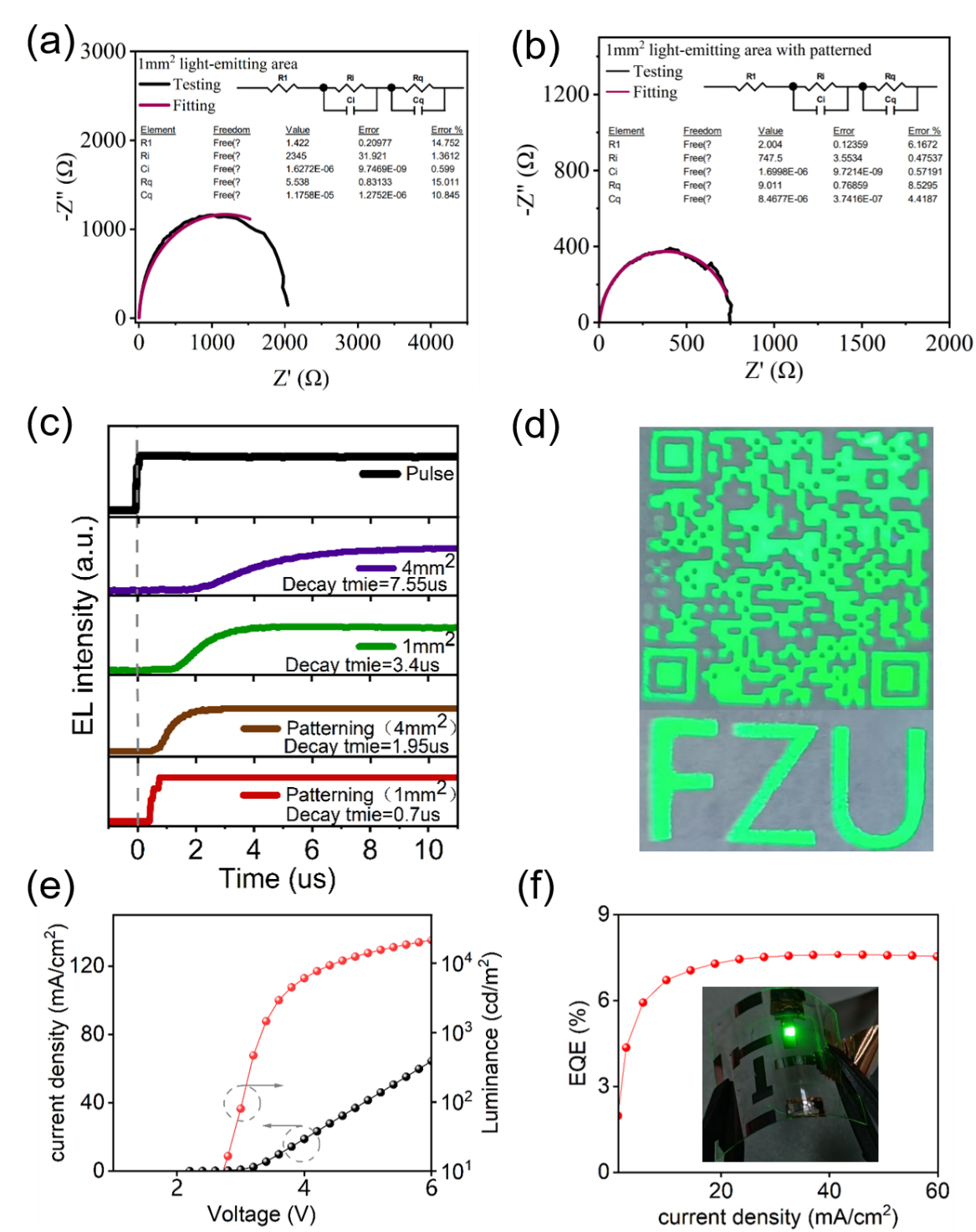


**Fig.S17.** (a-b) Impedance spectrum fitting results of 1 mm^2^ emitting area (without and with pixelation, respectively) devices. (c) TREL rising edge of devices with different light-emitting unit areas. (d) Two-dimensional code and FZU pattern of Fuzhou University official account transferred by seal. (e-f) The current density-voltage-luminance relationship curve and EQE curve of high-resolution flexible devices.


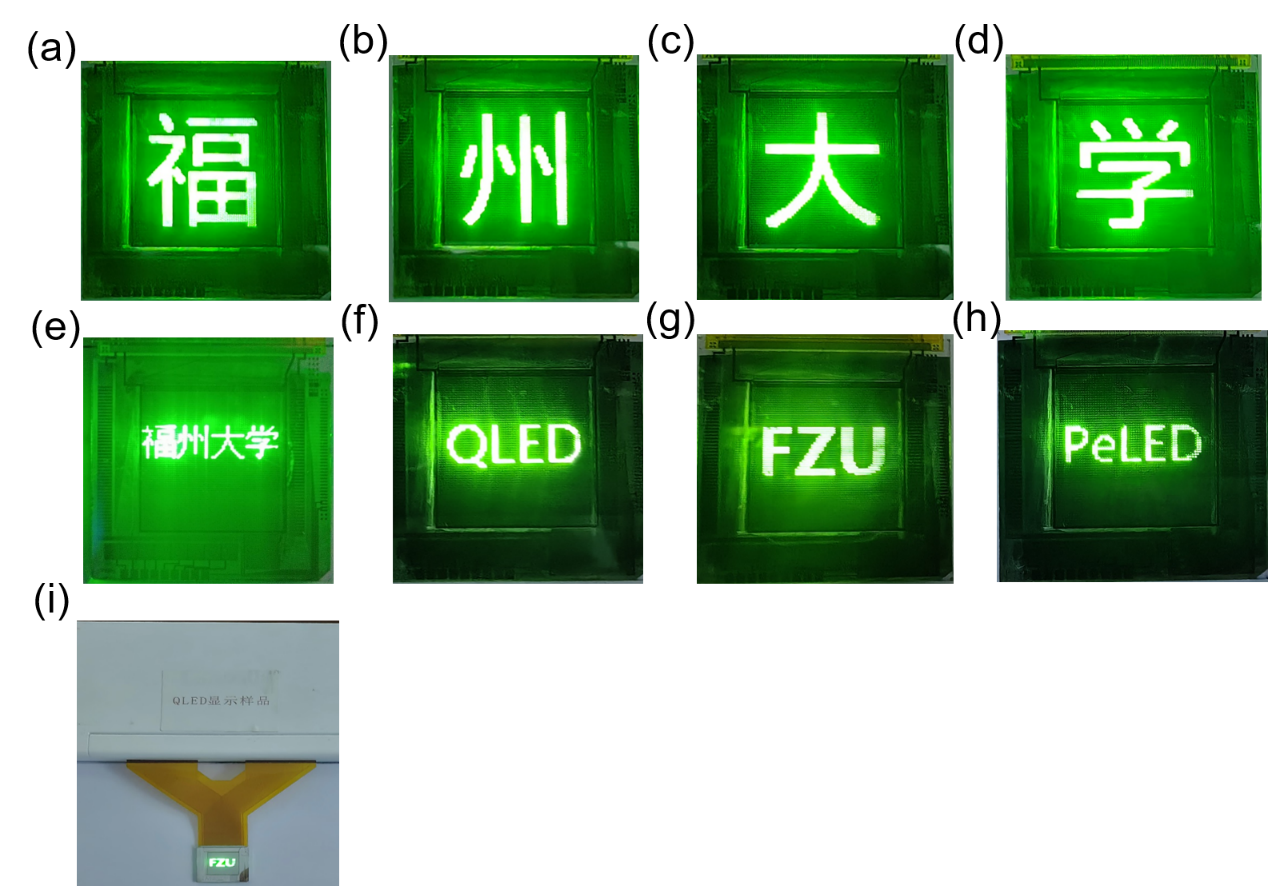


**Fig.S18.** (a-d) The Chinese name of Fuzhou University. (e-h) Different cartoon pictures of the character Xiao Zhi. (i) Test image of AM-PeLED displaying the letter of FZU.

**Table S1.** Exciton recombination lifetime of QDs.

|  | Control | [BMIM]OTF-1 | [BMIM]OTF-2 | [BMIM]OTF-3 |
| --- | --- | --- | --- | --- |
| A1 | 2.037E+3 | 1.768E+3 | 1.792E+3 | 1.782E+3 |
| A2 | 6.529E+2 | 8.905E+2 | 9.308E+2 | 9.799E+2 |
| A3 | 5.669E+1 | 8.914E+1 | 9.997E+1 | 9.596E+1 |
| **_τ_**_1_ | 1.895E-9 | 3.304E-9 | 2.884E-9 | 2.983E-9 |
| **_τ_**_2_ | 8.917E-9 | 1.474E-8 | 1.374E-8 | 1.327E-8 |
| **_τ_**_3_ | 4.511E-8 | 6.898E-8 | 6.878E-8 | 6.469E-8 |
| **_τ_** _ave_ | 14.26 | 25.36 | 26.72 | 29.84 |

By the three-index formula to fit and calculate average decay lifetime τ*_ave_*:

$I\left( t \right)=A1\exp\left( \frac{-t}{\boldsymbol{\tau}1} \right)+A2\exp\left( \frac{-t}{\boldsymbol{\tau}2} \right)+A3\exp\left( \frac{-t}{\boldsymbol{\tau}3} \right)$ (4)
$\boldsymbol{\tau}ave=\frac{{A1\tau1}^{2}+{A2\tau2}^{2}+{A3\tau3}^{2}}{A1\tau1+A2\tau2+A3\tau3}$ (5)

As shown in the table, which exhibited three decay lifetimes of τ_1_, τ_2_, τ_3_ and three fitting factor A_1_, A_2_, A_3_ [^1^]. Average decay lifetime τ*_ave_* is calculated by formula 2.

**Table S2.** Summary of recent research on patterning of QDs.

| Fabrication method | QD  materials | Resolution ratio  (PPI) | Peak EQE (%) | Peak Luminance  (cd m^-2^) | Year/Ref. |
| --- | --- | --- | --- | --- | --- |
| Photolithograpy | FAPbBr_3_ | 10*50 um | 6.8 | ＞20000 | 2022, Ref.[2] |
|  | FAPbBr_3_ | 4000 | 16 | 64726 | 2024, Ref.[3] |
| Inkjet printing | FA_0.3_ Cs_0.7_ PbBr_3_ | 120 | 2.8 | 1233 | 2020, Ref.[4] |
|  | CsPbBr_3_ | 250 | 8.54 | 43883 | 2022, Ref.[5] |
|  | Sr/Zn-doped  CsPb(I/Br)_3_ | 120 | 9.6 | 131 | 2022, Ref.[6] |
| Transfer printing | FA_0.15_ Cs_0.85_PbBr_3_ | 9072 | 7.81 | 146400 | 2023, Ref.[7] |
|  | [BMIM]OTF-  FA_0.15_Cs_0.85_PbBr_3_ | 9072 | 15.79 | 171547.13 | This work |

**Table S3.** Brightness, EQE and steady-state EL response time of PeLEDs in recent reports.

| Materials | Peak Luminance  (cd m^-2^) | Peak EQE (%) | Response time (μs) | Year/Ref. |
| --- | --- | --- | --- | --- |
| Perovskite  Film | 3035 | <1 | 6 | 2018, Ref. [8] |
|  | 83561 | 26 | 373 | 2023, Ref. [9] |
|  | 1806 | 9.25 | 1.3 | 2024, Ref. [10] |
| Perovskite  QDs | <10000 | 26.2 | >10 | 2024, Ref. [11] |
|  | 73784 | 20.94 | 3.4 | This work (N-HR) |
|  | 171547 | 15.79 | 0.7 | This work (HR) |

References

1. Zhang, J. *et al.* Highly Luminescent and Stable CsPbI_3_ Perovskite Nanocrystals with Sodium Dodecyl Sulfate Ligand Passivation for Red-Light-Emitting Diodes. *The Journal of Physical Chemistry Letters* **12**, 2437-2443, doi:10.1021/acs.jpclett.1c00008 (2021).

2． Dan Liu, K. W., Shaoyong Lu, Fu Li, Hannikezi Abudukeremu, , Lipeng Zhang，Yuchen Yang, J. H., Hengwei Qiu, Zhong Fu, Xiyu Luo & Lian DuanYouyu Zhang, H. Z., Jinghong Li. Direct optical patterning of perovskite nanocrystalswith ligand cross-linkers. *Science Advances* **8**, doi:10.1126/sciadv.abm8433 (2022).

3． Liu, D. *et al.* Nondestructive Direct Optical Patterning of Perovskite Nanocrystals with Carbene-Based Ligand Cross-Linkers. *ACS Nano* **18**, 6896-6907, doi:10.1021/acsnano.3c07975 (2024).

4． Li, D. *et al.* Inkjet Printing Matrix Perovskite Quantum Dot Light‐Emitting Devices. *Advanced Materials Technologies* **5**, doi:10.1002/admt.202000099 (2020).

5． Wei, C. *et al.* A Universal Ternary‐Solvent‐Ink Strategy toward Efficient Inkjet‐Printed Perovskite Quantum Dot Light‐Emitting Diodes. *Advanced Materials* **34**, doi:10.1002/adma.202107798 (2022).

6． Li, D. *et al.* Efficient red perovskite quantum dot light-emitting diode fabricated by inkjet printing. *Materials Futures* **1**, doi:10.1088/2752-5724/ac3568 (2022).

7． Mao, C. *et al.* Ultra‐High‐Resolution Perovskite Quantum Dot Light‐Emitting Diodes. *Advanced Optical Materials* **11**, doi:10.1002/adom.202202058 (2022).

8． Li, N., Lau, Y. S., Miao, Y. & Zhu, F. Electroluminescence and photo-response of inorganic halide perovskite bi-functional diodes. *Nanophotonics* **7**, 1981-1988, doi:10.1515/nanoph-2018-0149 (2018).

9． Li, Z. *et al.* Charge injection engineering at organic/inorganic heterointerfaces for high-efficiency and fast-response perovskite light-emitting diodes. *Nature Communications* **14**, doi:10.1038/s41467-023-41929-9 (2023).

10． Shen, C. *et al.* High performance and stable pure-blue quasi-2D perovskite light-emitting diodes by multifunctional zwitterionic passivation engineering. *Advanced Photonics* **6**, doi:10.1117/1.Ap.6.2.026002 (2024).

11． Gao, Y. *et al.* Microsecond-response perovskite light-emitting diodes for active-matrix displays. *Nature Electronics* **7**, 487-496, doi:10.1038/s41928-024-01181-5 (2024).
